# An atherosclerotic plaque-targeted single-chain antibody for MR/NIR-II imaging of atherosclerosis and anti-atherosclerosis therapy

**DOI:** 10.1186/s12951-021-01047-4

**Published:** 2021-09-28

**Authors:** Liwei Zhang, Sheng Xue, Feng Ren, Siyang Huang, Ruizhi Zhou, Yu Wang, Changyong Zhou, Zhen Li

**Affiliations:** 1 Institute for Translational Medicine, The Affiliated Hospital of Qingdao University, College of Medicine, Qingdao University, Qingdao, 266021 China; 2 Center for Molecular Imaging and Nuclear Medicine, State Key Laboratory of Radiation Medicine and Protection, School for Radiological and Interdisciplinary Sciences (RAD-X), Soochow University, Collaborative Innovation Center of Radiological Medicine of Jiangsu Higher Education Institutions, Suzhou, 215123 China; 3 Department of Radiology, The Affiliated Hospital of Qingdao University, Qingdao University, Qingdao, 266021 China

**Keywords:** atherosclerosis, oxidation-specific epitopes, single-chain variable fragment antibody, imaging, therapy

## Abstract

**Background:**

Oxidation-specific epitopes (OSEs) are rich in atherosclerotic plaques. Innate and adaptive immune responses to OSEs play an important role in atherosclerosis. The purpose of this study was to develop novel human single-chain variable fragment (scFv) antibody specific to OSEs to image and inhibit atherosclerosis.

**Results:**

Here, we screened a novel scFv antibody, named as ASA6, from phage-displayed human scFv library. ASA6 can bind to oxidized LDL (Ox-LDL) and atherosclerotic plaques. Meanwhile, ASA6 can also inhibit the uptake of Ox-LDL into macrophage to reduce macrophage apoptosis. The atherosclerotic lesion area of *ApoE*^−/−^ mice administrated with ASA6 antibody was significantly reduced. Transcriptome analysis reveals the anti-atherosclerosis effect of ASA6 is related to the regulation of fatty acid metabolism and inhibition of M1 macrophage polarization. Moreover, we conjugated ASA6 antibody to NaNdF_4_@NaGdF_4_ nanoparticles for noninvasive imaging of atherosclerotic plaques by magnetic resonance (MR) and near-infrared window II (NIR-II) imaging.

**Conclusions:**

Together, these data demonstrate the potential of ASA6 antibody in targeted therapy and noninvasive imaging for atherosclerosis.

**Graphic abstract:**

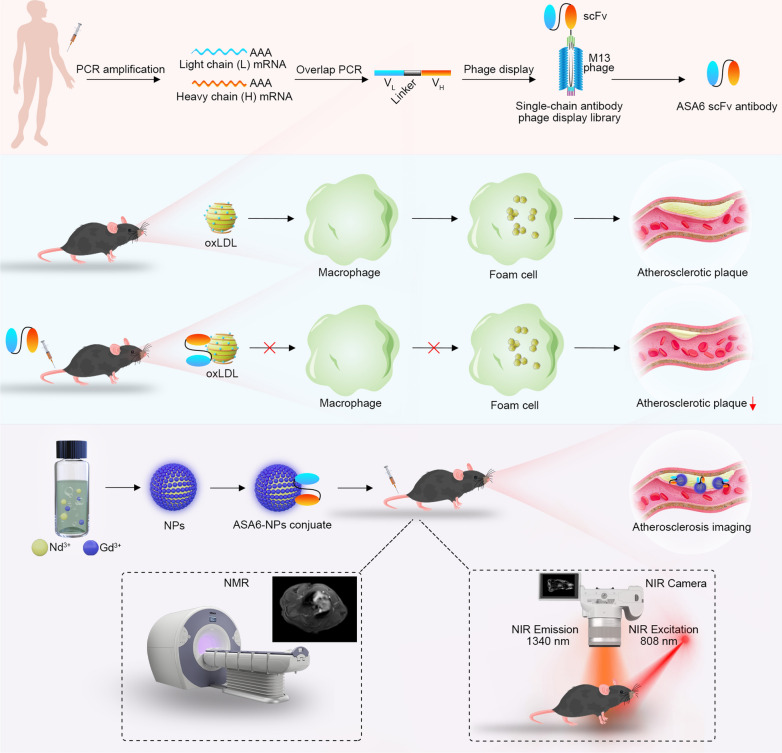

**Supplementary Information:**

The online version contains supplementary material available at 10.1186/s12951-021-01047-4.

## Background

Cardiovascular disease (CVD) is the leading cause of death worldwide which result in ≈ 17.8 million deaths globally, and the crude prevalence of CVD was 485.6 million cases in 2017 [1]. Atherosclerosis is a chronic inflammatory disease of arteries and is the most common underlying cause of CVDs [2]. Excessive low-density lipoprotein cholesterol (LDL-C) cause atherosclerosis, and research in the past decades have demonstrated that oxidized LDL (Ox-LDL) particles can promote atherogenesis [3, 4]. Native LDL can penetrate the intima and deposit in the arterial wall with macromolecules of intimal extracellular matrix when the barrier function of the endothelium is impaired [5]. Then the reactive oxygen species (ROS) in the subendothelial matrix could convert LDL into Ox-LDL, which is identified as a high cardiovascular risk factor and could promote the uptake of Ox-LDL into macrophages [6, 7]. The continuous engulfment of Ox-LDL mediated by scavenger receptors (e.g. CD36 and SRA1) leads to the transformation of macrophages into foam cells, which are the characteristic cells in the atherosclerotic lesions [8]. Therefore, Ox-LDL could induce chronic inflammation and eventually result in the initiation and progression of atherosclerotic lesions [9, 10].

Due to the complexity and heterogeneity of Ox-LDL, it harbours many modified lipid and neo-epitopes, such as oxidized phospholipids (OxPL) and malondialdehyde (MDA) modified apolipoprotein B-100 (apoB-100) epitopes, which are termed oxidation-specific epitopes (OSEs) [11]. OSEs could trigger innate and adaptive immunity, and IgM or IgG antibodies against oxidation epitopes in LDL were found in patients with atherosclerosis [12]. Previous studies showed that the level of OSEs specific IgM antibody was inversely associated with the incidence of carotid atherosclerosis and coronary artery disease (CAD) [13, 14]. The immunization against Ox-LDL may have an atheroprotective effect. A number of studies have shown that immunization of hypercholesterolemic rabbits and mice with Ox-LDL or apoB-100 peptides could impede the progression of atherosclerosis [15–17]. Previous studies have shown that specific antibodies recognizing OSEs can inactive Ox-LDL and block the uptake by macrophages, resulting in reducing foam cell formation and atherosclerosis progression. These OSEs specific antibodies are murine monoclonal antibodies [18, 19] or recombinant antibody fragments isolated from human antibody phage display library [20–23].

Phage display as a high-throughput in vitro display technique allows to obtain antibodies that would be extremely difficult, if not impossible, to generate by animal immunization [24]. In general, small antibody fragments (e.g. Fab and scFv) are smaller in size, lack the Fc region and possess unique tissue penetration properties suitable to therapeutic and imaging applications [25]. Previous studies have demonstrated that elevated levels of circulating OSEs is highly associated with presence and progression of CAD, so development of sensitive molecular imaging probes that target OSEs in the artery wall may enable the noninvasive detection of vulnerable plaques [26]. Due to the submillimeter resolution with unlimited penetration depth, magnetic resonance imaging (MRI) has been considered as a promising technique for noninvasive atherosclerotic lesion diagnosis by the direct assessment of plaque burden and composition [27, 28]. However, MRI is limited by long scanning and post-analyzing times, and the sensitivity is very low even with injected contrast. The fluorescence imaging in the second near-infrared window (NIR-II, 1000–1700 nm) has recently emerged as a promising noninvasive angiographic method with the advantages of fast imaging capability, deep tissue penetration, nonionizing radiation and low cost [29–33]. However, human coronary vessels NIR-II imaging will still be extremely challenging due to the limited penetration of NIR light in human tissue. We hypothesized that the conjugation of MRI/NIR-II molecular probes to OSEs specific antibodies will achieve accurate diagnosis of atherosclerotic lesions and will obtain the characterization of vulnerable plaque.

**Fig. 1:**
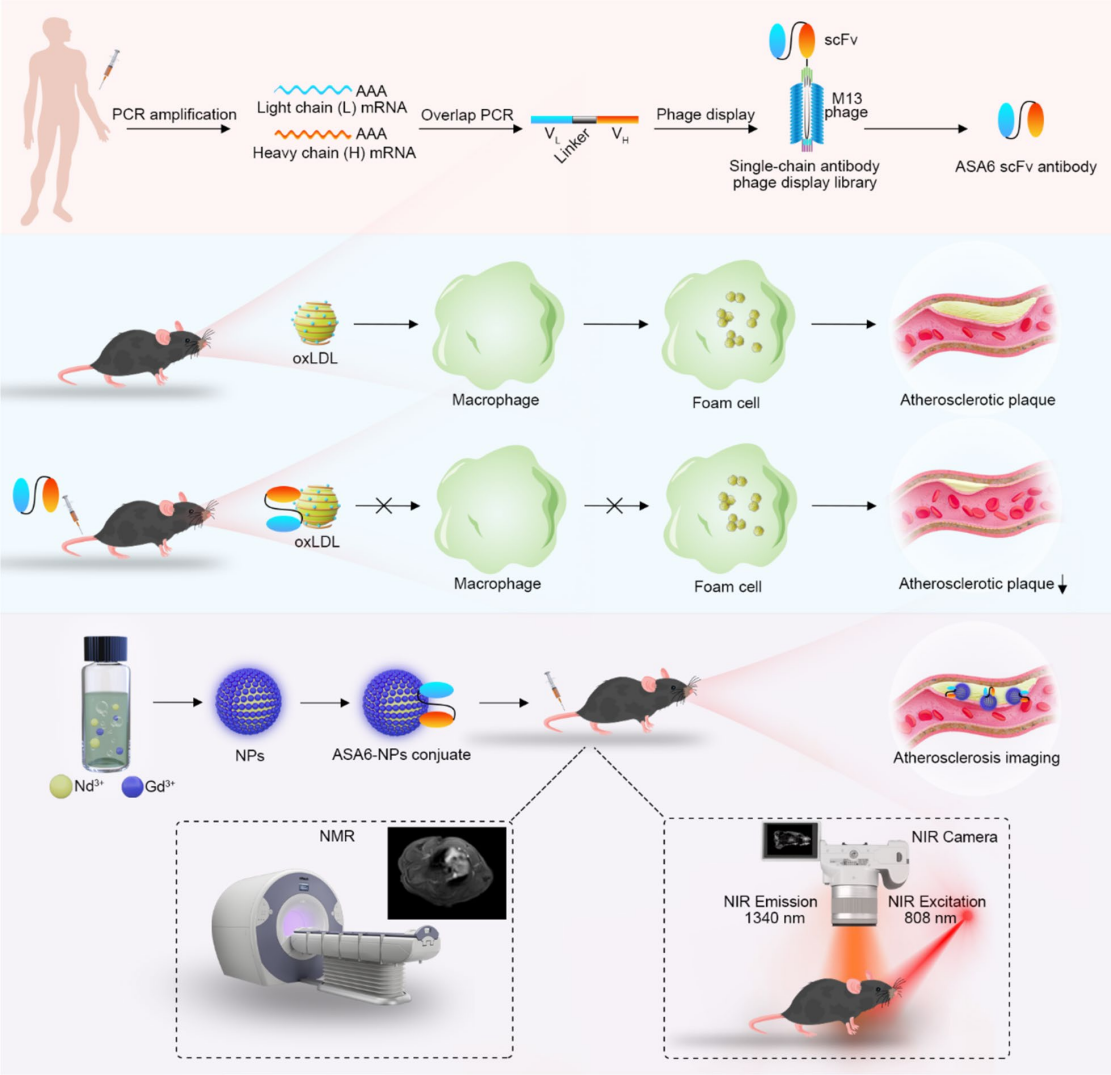
Schematic illustration of isolation of ASA6 and its application in imaging and treatment of atherosclerosis. ASA6 are screened from human antibody phage display library based on peripheral blood of CAD patients. ASA6 can specifically bind to Ox-LDL particles to inhibit the uptake of Ox-LDL by macrophages. ASA6 exhibits a therapeutic effect against atherosclerosis in *ApoE*^*−/−*^ mice. Due to the specific binding to atherosclerotic plaques, ASA6 can be used in MR/NIR-II imaging of atherosclerosis by conjugated with NaNdF_4_@NaGdF_4_ nanoparticles

Herein, we report a new human scFv antibody, named as ASA6, which was isolated by screen a human antibody phage display library against human atherosclerotic lesions (Fig. 1). We found ASA6 antibody could bind to Ox-LDL and inhibit the uptake of Ox-LDL by macrophages to reduce the apoptosis of macrophages. In addition, ASA6 antibody can decrease atherosclerotic plaque area in *ApoE*^−/−^ mice after in vivo treatment. The ASA6 antibody was then conjugated to MRI/NIR-II dual-function probe, and imaging efficacy for noninvasive detection of atherosclerotic plaques was investigated in present study.

## Materials and methods

### Animals

Male 6-week-old C57BL/6J mice (wild type, Vital River, Beijing, China) were used to evaluate biocompatibility of nanoparticles. Male 6-week-old *ApoE*^−/−^ mice (Vital River, Beijing, China) were fed with a high-fat diet (HFD) (containing 15% fat and 1.5% cholesterol) to induce atherosclerosis. All mice were maintained on 12-h dark/12-h light cycles with access to food and drinking water, *ad libitum.* Mice were euthanized by inhalation of 5 % isoflurane (RWD Life Science, Shenzhen, China) and cervical dislocation to harvest organs.

### Sample collection and RNA preparation

A total of 55 patients with angiographically defined coronary artery disease from the Affiliated Hospital of Qingdao University (Shinan District) were selected and total cholesterol (TG), total triglyceride (TC), high density lipoprotein (HDL) and low density lipoprotein (LDL) levels were measured (Additional file 1: Table S1). Then, arterial blood samples from these patients were collected, and lymphocytes were separated by using lymphocyte separation medium (Solarbio, Beijing, China). Total RNA was extracted from lymphocytes using the Trizol (Sigma-Aldrich, St Louis, MO, USA). Meanwhile, several human arteries with atherosclerotic plaques were obtained from division of vascular surgery of the Affiliated Hospital of Qingdao University (Shinan District).

### Construction of scFv phage display library

Construction and biopanning of scFv phage display library, identification of positive clones and scFv antibody purification was performed according to the methods of previously described [34, 35], with PCR primers listed in Additional file 1: Table S2.

### Immunofluorescence and oil-red O staining

A section of postmortem human atherosclerotic coronary artery was embedded in OCT and snap-frozen in liquid nitrogen. Series of 10-µm-thick slices were transversely sectioned. Non-specific binding sites were blocked by 3% BSA in PBS at 37 °C for 1 h. The slides were incubated with ASA6 (1 µg/mL) at 37 °C for 1 h. Then the slides were incubated with anti-His tag mouse mAb (1:5000) (Sino Biological, Beijing, China) at 37 °C for 1 h and 1:100 diluted Cy3-labeled Goat anti-Mouse IgG (H + L) (ABclonal, Wuhan, China) at 37 °C for 1 h. DAPI was used for nuclear staining. The images were captured using Nikon Eclipse Ti fluorescence microscope (Nikon, Tokyo, Japan). Percentage of positively stained areas within the lesion were evaluated by Image J software 1.52 (National Institutes of Health, Bethesda, MD, USA). Meanwhile, the atherosclerotic lesions were stained by oil-red O and photographed.

### Sequencing analysis of ASA6

The gene coding sequence of ASA6 was obtained using forward primer ompseq (5′-AAGACAGCTATCGCGATTGCAG-3′) and backward primer gback (5′-GCCCCCTTATTAGCGTTTGCCATC-3′), and the amino acid sequence of ASA6 was analyzed with NCBI BlastP online tool.

### Synthesis of ASA6 antibody-conjugated NaNdF_4_@NaGdF_4_ nanoparticles (ASA6-NPs)

Step 1: preparation of NaLnF_4_ (Ln = Nd, Gd) nanoclusters.

An amount of 1 mL of neodymium (III) chloride hexahydrate solution (NdCl_3_, 0.5 M) and 4 mL of sodium fluoride solution (NaF, 0.5 M) were added dropwise to the homogeneous solution including 1.2 g sodium hydroxide (NaOH), 9 mL of absolute ethanol, 4 mL of deionized water and 20 mL of oleic acid. After stirring at RT for 1 h, α-NaNdF_4_ nanoclusters were precipitated by 35 mL of absolute ethanol. The obtained α-NaNdF_4_ nanoclusters were washed twice by absolute ethanol, and finally dispersed in 2 mL of cyclohexane for further use.

Step 2: synthesis of β-NaNdF_4_@β-NaGdF_4_ core-shell nanoparticles (NPs).

An amount of 6 mL of oleic acid, 10 mL of 1-octadecene, and 2 mL of α-NaNdF_4_ nanoclusters cyclohexane solution (0.25 M) were added successively in a 100 mL of three-neck flask. To ensure that cyclohexane is completely removed, the mixture was stirred under a stream of nitrogen at 70 °C. The temperature was then raised to 280 °C at a rate of ~ 15 °C/min, and the temperature was lowered to 70 °C after 30 min of reaction at 280 °C. After adding NaGdF_4_ nanocluster solution (0.5 mM), β-NaGdF_4_ shell growth was performed according to the same reaction conditions of β-NaNdF_4_ core nanocrystals. After the reaction, the temperature was cooled to 70 °C, and then NaGdF_4_ nanocluster solution (0.25 mM) was added, and the shell thickness was increased according to the same shell reaction conditions. Finally, the reaction temperature was cooled to RT, and the reaction production was obtained after the centrifugation at 11,000 rpm for 10 min. After washing twice by absolute ethanol, the product was dispersed in chloroform.

Step 3. Typically, 10 mg NPs were mixed with phosphorylated brush polymer (50 mg) with overnight stirring. Then, the polymer coated nanoparticles were precipitated by cyclohexane and dried under a vacuum at room temperature. The dried nanoparticles were redispersed in the saline solution (5 mL), and further purified by ultrafiltration at 1500×*g* for 3 times to remove free polymer.

Step 4. ASA6 was conjugated to NPs by EDC/NHS reaction. Firstly, 5 mg of NaNdF_4_@NaGdF_4_ nanoparticles were concentrated by ultrafiltration at 1500×*g* for 8 min, then the NPs were resuspended by 600 µL of PBS buffer (pH 6.8). Then, 10 mg of EDC and 5 mg of NHS dissolved in 200 µL of PBS (pH 6.8) were add to the solution above and shake at 150 rpm for 30 min for activating the carboxyl group. The activated NPs were washed twice with PBS buffer (pH 7.2) by ultrafiltration and resuspended in 1 mL of PBS buffer (pH 7.2). 1 mL of ASA6 (3.67 mg/mL) in PBS buffer (pH 7.2) was added to the solution with EDC/NHS activated NPs and shaken at RT for 4 h. After conjugation, the ASA6-NPs probe was washed five times by PBS buffer (pH 7.2) and resuspended in 1 mL of PBS buffer (pH 7.2) with 0.05 % (w/v) NaN_3_ and 1 % (w/v) BSA and store at 4 °C. TEM images were captured with a FEI Tecnai G20 transmission electron microscope (FEI, Hillsboro, USA) operating at an acceleration voltage of 120 kV. The hydrodynamic sizes of NPs and ASA6-NPs were tested at 25 °C using Malvern Zetasizer Nano ZS90 with dynamic light-scattering (DLS) (Malvern, Worcestershire, UK) with a solid-state He–Ne laser (λ = 633 nm).

### The biocompatibility assessment of ASA6-NPs probe in vitro

The cytotoxicity of ASA6-NPs was determined by the MTT assay in vitro. In brief, human aortic endothelial cells (HAECs) were seeded into 96-well culture plates and incubated at 37 °C in an incubator with 5% CO_2_ for 24 h. Then, the cell culture medium was aspirated and fresh culture media containing various concentrations of ASA6-NPs (0.16, 0.32, 0.65, 1.30 mg/mL) were added. After 6, 12 and 24 h, relative cell activity was analyzed by MTT assay kit (Meilun, Dalian, China).

### The biocompatibility assessment of ASA6-NPs probe in vivo

Male 6-week-old C57BL/6J mice were divided into two groups randomly: control group and ASA6-NPs injection group, each group containing 5 mice. Mice in ASA6-NPs treated group were injected with 100 µL of ASA6-NPs (Gd concentration of 1.39 mg/mL) per mouse via the tail vein, while mice in the control group were injected with 100 µL PBS buffer via the tail vein. After seven days, mice were sacrificed to harvest major organs (heart, liver, kidney, spleen, bladder, cerebral cortex and hippocampus) and blood samples. For H&E staining, organs were fixed with 4% paraformaldehyde for 24 h. The total RNA from liver and kidney tissue were extracted using Trizol Reagent and cDNA was synthesized. Gene expression levels of IL-6, IL-10, IL-11a, IL-11b, MCP-1 and TNF-α in liver and kidney were quantified by TransStart^®^ Green qPCR SuperMix (Transgen, Beijing, China) by normalized to GAPDH using comparative threshold (*F* = 2^−∆∆Ct^) method. Serum CRP, IL-6 and TNF-α levels were quantified by ELISA assay kit (Boster, Wuhan, China). Kidney tissues were performed chlorophosphonazo III staining to analyze the distribution of ASA6-NPs nanoparticles [36]. The RGB values of chlorophosphonazo III staining were analyzed by Matlab software.

### MR/NIR-II imaging for atherosclerotic plaque in vivo

Male 6-week-old *ApoE*^−/−^ mice were purchased from Vital River (Beijing, China). *ApoE*^−/−^ mice were fed with a high-fat diet (HFD) (containing 15 % fat and 1.5 % cholesterol) for 12 weeks. After 12 weeks, mice (n = 3) were injected with 100 µL of ASA6-NPs (Gd concentration of 1.39 mg/mL) through the tail vein for MR/NIR-II imaging at different time points. NIR-II imaging of atherosclerotic lesions were captured by NIR-II imaging system (Serious II 900–1700 nm) (Suzhou NIR-Optics Co., Ltd., China). The power density of 808 nm laser was set to 150 mW/cm^2^ for 1340 nm emission. The quantitative analysis of the images was performed by Image J software.

For MR imaging of atherosclerotic lesions, T_1_-weighted images *ApoE*^−/−^ mice (n = 3) were captured on MAGNETOM PRISMA 3T (SIEMENS, Erlangen, Germany) using a 3D free-breathing STAR VIBE sequence with the following parameters: TE, 2.91 ms; TR, 5.72 ms; flip angle, 20°; spatial resolution, 0.30 × 0.30 mm^2^; slide thickness = 1.5 mm. MRI images were analyzed by Syngo FastView (SIEMENS, Germany) and Image J software. After imaging, mice were sacrificed and whole aorta were isolated. For en face analysis of lesions, the entire aorta from the aorta root to the iliac arteries was opened longitudinally and stained with Oil Red O. Lesions were quantified by morphometry of images using Image J software.

### In vitro protective effect of ASA6 against atherosclerosis

The inhibition of Ox-LDL induced apoptosis by ASA6 was evaluated by the TUNEL assay. RAW 264.7 macrophages were divided into five groups: control group (RAW 264.7 macrophages), Ox-LDL group (RAW 264.7 macrophages + 50 µg/mL Ox-LDL), ASA6 group (RAW 264.7 macrophages + 100 µg/mL ASA6), ASA6 + Ox-LDL group 1 (RAW 264.7 macrophages + 50 µg/mL ASA6 + 50 µg/mL Ox-LDL), ASA6 + Ox-LDL group 2 (RAW 264.7 macrophages + 100 µg/mLASA6 + 50 µg/mL Ox-LDL). In brief, RAW 264.7 macrophages were seeded into 24-well culture plates and incubated at 37 °C in an incubator with 5% CO_2_ for 24 h. The cell culture medium was aspirated and fresh culture media containing 1% FBS and various concentrations of pre-mixed Ox-LDL and ASA6 mixture were added. After 24 h, cell apoptosis was analyzed by TUNEL assay kit (Yeasen, Shanghai, China).

The inhibition of Ox-LDL uptake into macrophages by ASA6 was assessed using Dil-Ox-LDL (Yeasen, Shanghai, China). RAW 264.7 macrophages were divided into three groups: control group (RAW 264.7 macrophages), Dil-Ox-LDL group (RAW 264.7 macrophages + 40 µg/mL Dil-Ox-LDL), ASA6 + Dil-Ox-LDL group (RAW 264.7 macrophages + 50 µg/mL ASA6 + 40 µg/mL Ox-LDL). In brief, RAW 264.7 macrophages were seeded into 24-well culture plates and incubated at 37 °C in an incubator with 5% CO_2_ for 24 h. The cell culture medium was aspirated and fresh culture media containing 1% FBS and various concentrations of pre-mixed Dil-Ox-LDL and ASA6 mixture were added. After 6 h, cells were washed three times by PBS buffer then fixed in 4 % paraformaldehyde for 30 min at RT. After washing, DAPI was used for nuclear staining. The experiments were repeat three times, and positive cells were quantified by Image J software.

### The assessment of anti-atherosclerotic effect by ASA6 in vivo

Male 6-week-old *ApoE*^−/−^ mice were fed with a high-fat diet (HFD) (containing 15 % fat and 1.5 % cholesterol) for 12 weeks. *ApoE*^−/−^ mice were divided into two groups (control group and ASA6 group), each group containing 10 mice. *ApoE*^−/−^ mice in ASA6 group were intraperitoneally administrated with ASA6 at a dose of 2.5 mg/kg twice a week when starting with HFD, and mice in control group were intraperitoneally administrated with PBS buffer twice a week, respectively. After 12 weeks, mice were sacrificed to harvest heart and aorta. After fixation with 4 % paraformaldehyde, heart was embedded in OCT compound, and serial frozen sections (10 μm) of aortic root were obtained. Atherosclerotic lesions in aortic sinus were stained by Oil Red O. Percentage of the lesion area in the intima was analyzed by Image J software.

The aorta was subjected to transcriptome analysis. Total RNA of aorta tissues was extracted using Trizol reagent (Sigma-Aldrich, St Louis, MO, USA) according to RNA extract protocol, and were used to construct sequencing library. The RNA purification, reverse transcription, library construction, and sequencing were performed at Majorbio Bio-pharm Biotechnology Co., Ltd (Shanghai, China) using Illumina HiSeq X10 (Illumina, San Diego, CA, USA) according to the manufacturer’s instructions. RNA-seq transcriptome library was prepared following TruSeqTM RNA sample preparation Kit from Illumina (San Diego, CA, USA) using 1 µg of total RNA. After quantified by TBS380, paired-end RNA-seq sequencing library was sequenced with the Illumina HiSeq xten/NovaSeq 6000 sequencer (2 × 150 bp read length). The raw paired end reads were trimmed and quality controlled by SeqPrep (https://github.com/jstjohn/SeqPrep) and Sickle (https://github.com/najoshi/sickle) with default parameters. Then clean reads were separately aligned to reference genome with orientation mode using TopHat (http://tophat.cbcb.umd.edu/, version2.0.0) [37] software.

To identify DEGs (differential expression genes) between two different samples, the expression level of each transcript was calculated according to the fragments per kilobase of exon per million mapped reads (FRKM) method. RSEM (http://deweylab.biostat.wisc.edu/rsem/) [38] was used to quantify gene abundances. R statistical package software EdgeR (Empirical analysis of Digital Gene Expression in R, (http://www.bioconductor.org/packages/2.12/bioc/html/edgeR.html) [39] was utilized for differential expression analysis. In addition, functional-enrichment analysis including GO and KEGG were performed to identify which DEGs were significantly enriched in GO terms and metabolic pathways at Bonferroni-corrected P-value ≤ 0.05 compared with the whole-transcriptome background. GO functional enrichment and KEGG pathway analysis were carried out by Goatools (https://github.com/tanghaibao/Goatools) and KOBAS (http://kobas.cbi.pku.edu.cn/home.do) [40].

### Statistical analysis

The results were expressed as means ± SD of at least three independent experiments. The differences among experimental groups were evaluated by one-way ANOVA or Student’s *t* test analysis of variance. *p* < 0.05 was considered statistically significant. GraphPad Prism-8 statistic software (La Jolla, CA, USA) was used for all data analysis.

## Results

### The construction of scFv library and biopanning

Total cellular RNA was extracted from human peripheral blood lymphocyte of patients with myocardial infarction from the Affiliated Hospital of Qingdao University (Shinan District). And then mRNA was purified and transcribed into cDNA for antibody variable fragment amplification. Single-chain variable fragment (scFv) phage display library was constructed with a size of 2.5 × 10^7^ clones. The phage library was then screened against human atherosclerosis plaque through three rounds of panning. The input of phages in each round panning were approximately 10^12^ and the output ranged from 10^4^ to 10^6^. The ratios of output to input phages increased 10^2^-fold after three rounds of panning (Additional file 1: Table S3), indicating that the phages targeted to atherosclerotic plaque were enriched successfully. To isolate scFv antibodies with high affinity, 24 phage clones were randomly selected from the output plate of the last round and screened by phage-ELISA. There were 12 phage clones exhibited high affinity for atherosclerotic plaques compared to BSA control, with one phage clone, named as ASA6, showing the strongest affinity (Additional file 1: Fig. S1).

### Amino acid sequence analysis of scFv antibody

**Fig. 2:**
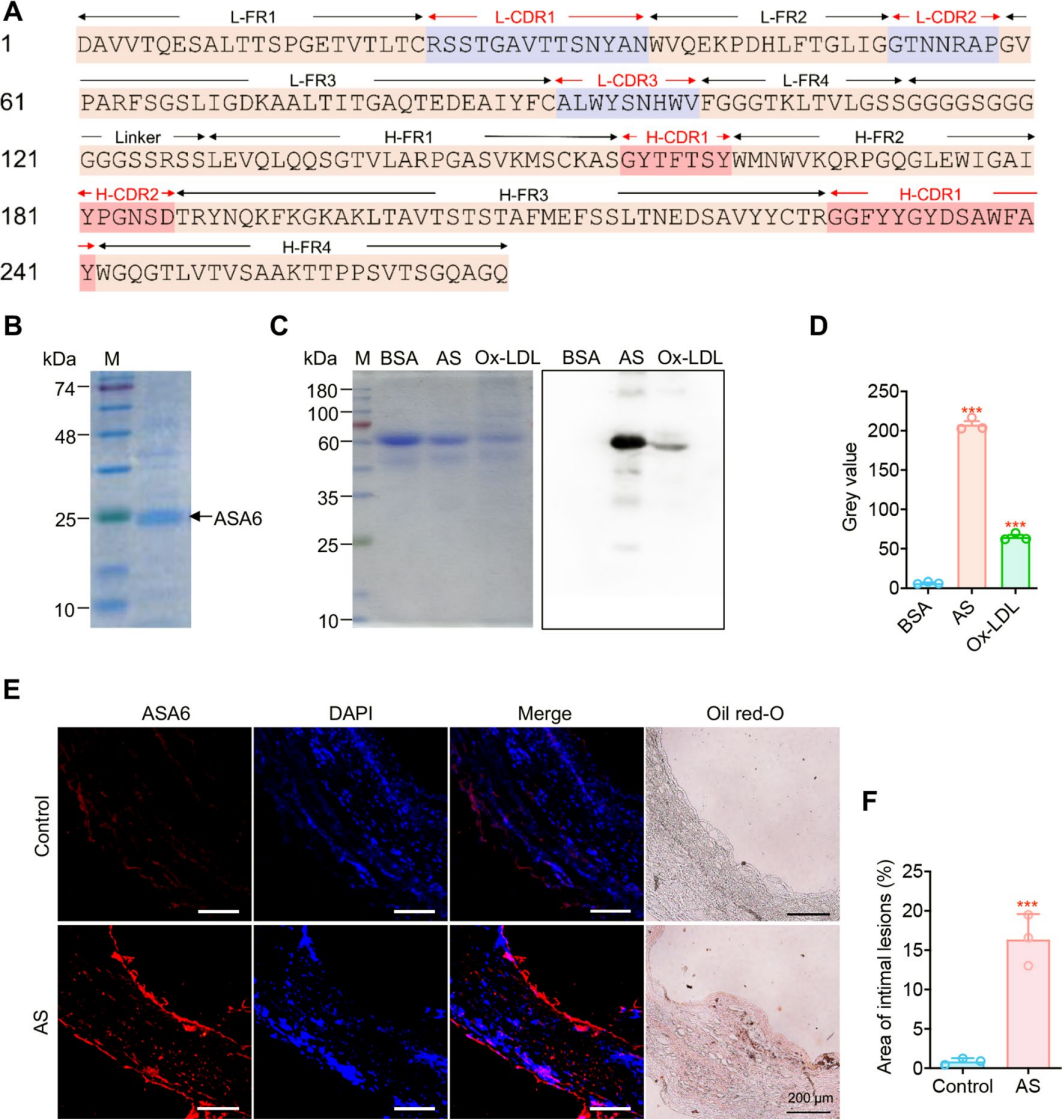
Identification and characterization of ASA6. **A** Amino acid sequences of complimentary determining region of heavy chain (CDR-H) and light chain (CDR-L) of ASA6 antibody. **B** Image of a Coomassie brilliant blue-stained SDS-PAGE gel of purified ASA6 antibody. **C** Immunoblots of protein extract from human aortic plaque (AS) and Ox-LDL detected with ASA6 antibody. BSA control, AS and Ox-LDL were separated by SDS-PAGE (left), and blotted onto nitrocellulose membranes (right). **D** The quantitative analysis of Immunoblots. **E** Detection of atherosclerotic lesions in human coronary arteries by immunofluorescence with ASA6 antibody and Oil-red O staining. **F** The quantitative analysis of immunofluorescence staining. Data are represented as means ± s.d. from three independent replicates. ^***^p < 0.001, one-way ANOVA (G) or Student’s *t* test (I)

The deduced amino acid sequences of ASA6 are shown in Fig. 2A. The framework regions (FRs) and complementary-determining regions (CDRs) in the V_H_ and V_L_ chains of ASA6 were predicted by IgBlast database (https://www.ncbi.nlm.nih.gov/igblast) and the result showed that ASA6 have complete human V_H_ and V_L_ domain (Fig. 2A), and the amino acid sequences of ASA6 was never reported previously.

### Expression, purification and characterization of ASA6

To prepare soluble ASA6 antibody, pComb3XSS-ASA6 phagemid was transformed into *E. coli* TOP10. After IPTG-induced expression and purification by Ni-NTA agarose, the concentration of soluble antibody was about 350 µg/mL. After further concentration by ultrafiltration, the concentration of soluble antibody was 7 mg/mL. Meanwhile, purified ASA6 antibody was identified by SDS-PAGE with a molecular weight about 25 kDa (Fig. 2B).

In order to identify the interaction between ASA6 and human atherosclerotic plaques, we performed western blot (Fig. 2C and D). The result showed that ASA6 antibody strongly bound to the protein components of human atherosclerotic plaques and Ox-LDL (Fig. 2C). The grey value analysis of western blot showed ASA6 antibody binding to human atherosclerotic plaques and Ox-LDL was statistically higher than to BSA control (*p* < 0.001) (Fig. 2D). These results showed that ASA6 can specifically recognize atherosclerotic plaques and Ox-LDL which is present within atherosclerotic lesions of arteries. In order to prove the antigen-antibody affinity at the histological level, we performed immunofluorescence experiment and Oil-red O staining using human atherosclerotic artery segments. Atherosclerotic lesions were stained red by Oil-red O and the immunofluorescence signals demonstrated that ASA6 can recognize atherosclerotic lesions, which enriched in intima of the artery (Fig. 2E). Due to normal artery tissue also has some component similar to the lesion, there was very weak fluorescence signal in normal control. However, the statistical analysis of immunofluorescence signals showed there was statistically significant difference (*p* < 0.001) between normal and atherosclerotic artery (Fig. 2F). The marked area in atherosclerotic plaque is much more than that in normal artery tissue. Thus, we have evidence that ASA6 can target to atherosclerotic plaques, as well as Ox-LDL *in vivo* within atherosclerotic lesions.

### Synthesis of ASA6 conjugated nanoparticle (ASA6-NPs) probe

**Fig. 3:**
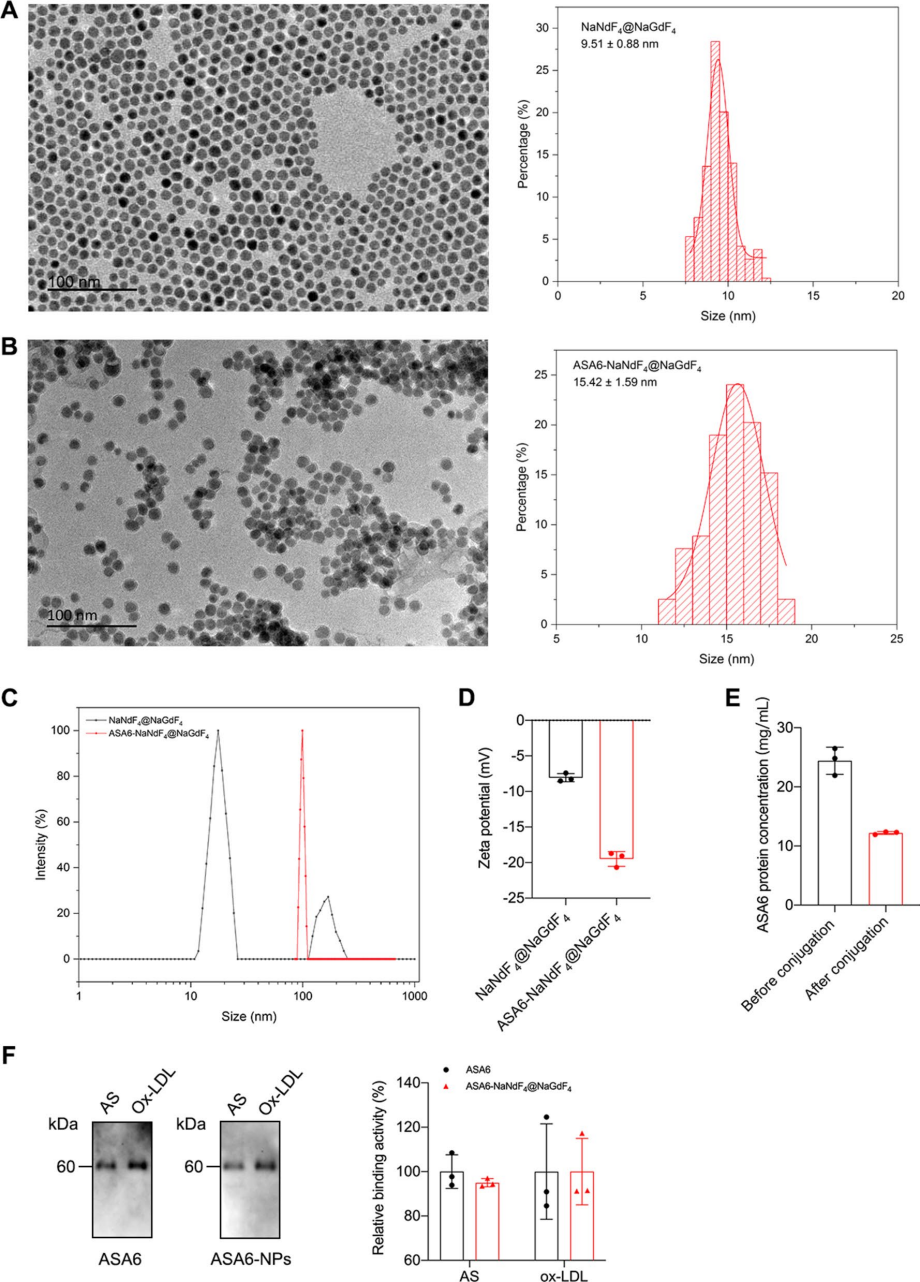
Evaluation of ASA6 conjugated nanoparticles. **A** TEM images and size histograms of NaNdF_4_@NaGdF_4_ nanoparticles. **B** TEM images and size histograms of ASA6-NaNdF_4_@NaGdF_4_ nanoparticles. **C** Hydrodynamic size distribution profiles, and zeta potential histogram (**D**) of NaNdF_4_@NaGdF_4_ and ASA6-NaNdF_4_@NaGdF_4_ nanoparticles. **E** Amount of ASA6 conjugated to NaNdF_4_@NaGdF_4_ nanoparticles. **F** Binding capability of ASA6 to target before and after conjugation

Mixed NdCl_3_ and NaF with homogeneous solution including NaOH, absolute ethanol, deionized water and oleic acid leaded to the production of α-NaNdF_4_ nanoclusters, which can be used as near-infrared (NIR) probe with good photostability after high-temperature treatment for nanocrystal growth. To make nanoparticles have the contrast-enhancing performance for MRI and improve the second near-infrared window (NIR-II, 1000–1700 nm) imaging ability, NaGdF_4_ was selected to coat NaNdF_4_ cores to achieve high performance MRI/NIR-II bifunctional nanoparticles. As shown in Fig. 3A, NaNdF_4_@NaGdF_4_ nanoparticles were highly monodisperse with an average size of 9.51 ± 0.88 nm. The NaNdF_4_@NaGdF_4_ nanoparticles also showed good absorption with main absorption peaks at 574 nm, 741 nm, 795 nm and 865 nm (Additional file 1: Fig. S2). The NIR-II fluorescence spectrum showed a maximum NIR-II emission peak at 1059 nm (Additional file 1: Fig. S3). The longitudinal relaxivity against the concentrations of RE^3+^ ions (Nd^3+^ + Gd^3+^) under 3 Tesla magnetic field was measured to be 4.27 mM^− 1^ S^− 1^ (Additional file 1: Fig. S4). To detect atherosclerosis by targeted MR/NIR-II imaging, ASA6 was conjugated to NaNdF_4_@NaGdF_4_ nanoparticles by EDC-NHS reaction to form ASA6-NPs probe. As Fig. 3B shown, this conjugate of ASA6 and NaNdF_4_@NaGdF_4_ nanoparticles was highly monodisperse, and the average size increased to 14.42 ± 1.59 nm with a low-density layer also observed on ASA6-NaNdF_4_@NaGdF_4_ surface under transmission electron microscopy. The hydrodynamic size of ASA6-NaNdF_4_@NaGdF_4_ (129.99 ± 2.35 nm) was reasonably larger than NaNdF_4_@NaGdF_4_ (21.76 ± 0.14 nm) due to surface attachment of antibody molecular (Fig. 3C). After conjugation reaction, the surface zeta potential of nanoparticles was changed from − 8.06 ± 0.34 mV to − 19.49 ± 0.60 mV (Fig. 3D). The ASA6 protein concentration on nanoparticles was 12.21 mg/mL in solution, which demonstrated 50 % of ASA6 protein was conjugated to NaNdF_4_@NaGdF_4_ particles (Fig. 3E). Immuno-blot assay proved ASA6-NaNdF_4_@NaGdF_4_ (ASA6-NPs) retained 94.98 % and 95.36 % binding activity to the target of human aortic plaque and Ox-LDL protein compared with unconjugated ASA6 (Fig. 3F). These results supported the successful formation of ASA6-NaNdF_4_@NaGdF_4_ conjugate.

### The biocompatibility of ASA6-NPs probe

**Fig. 4:**
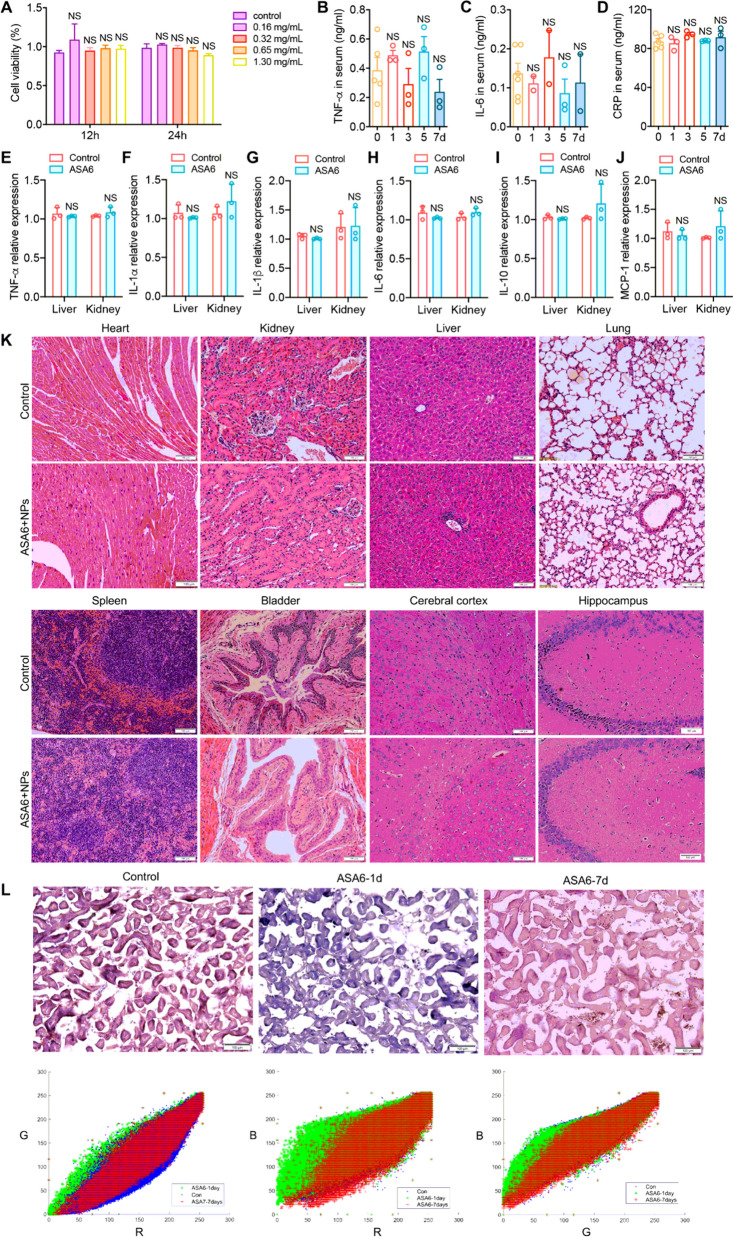
In vitro and in vivo biocompatibility assessment of ASA6-NPs probe. **A** The MTT assays of HAECs after incubated with ASA6-NPs probe for 12 and 24 h. **B**–**D** The serum levels of inflammation factors tumor necrosis factor-α (TNF-α), interleukin-6 (IL-6) and C-reactive protein (CRP) in different time points after the probe injection in mouse. **E**–**J** Mouse mRNA expression levels of inflammatory cytokines TNF-α, IL-1α, IL-1β, IL-6, IL-10 and monocyte chemoattractant protein-1 (MCP-1) in liver and kidney at 7 days post-injection. **K** The HE staining (including heart, kidney, liver, lung, spleen, bladder, cerebral cortex and hippocampus) of mice at 7 days post-injection. **L** The chlorophosphonazo III staining of mouse kidney at 1 day and 7 days after injection, and their RGB value analysis. Data represent means ± s.d. from three independent replicates. NS, no significance as indicated, one-way ANOVA

Firstly, we evaluated the toxicity of ASA6-NPs probe to the human aortic endothelial cells (HAEC) *in vitro*, for the reason that the probe was directly injected into circulation system during targeted MR/NIR-II imaging. We used Gd^3+^ concentration to represent the concentration of ASA6-NPs probe. HAECs were exposed to various concentration of ASA6-NPs (0, 0.1625, 0.325, 0.65, 1.3 and 2.6 mg/mL) and in different exposure time (12 and 24 h). Cell viability was analyzed by MTT assay, and the result showed no significant difference in cell viability between control and ASA6-NPs treated cells (Fig. 4A). This result suggesting that ASA6-NPs probe have no obvious cytotoxicity effect on endothelial cells.

Then, we tested the biocompatibility of ASA6-NPs probe in vivo. To assess inflammatory response in mice intravenously administrated with the probe (Gd^3+^ concentration 4.3 mg/kg, equal to 0.13 mg/mL for a 30 g mouse with 1 mL of blood), serum cytokine levels, inflammatory factor expression and major organ histopathology were examined at 7 days after intravenous administration of ASA6-NPs. As is shown in Fig. 4B–D, there were no significant difference in serum levels of tumor necrosis factor-α (TNF-α), interleukin-6 (IL-6) and C-Reactive protein (CRP) after administration of ASA6-NPs, suggesting that no obvious inflammatory responses were triggered, particularly in acute inflammatory responses. Meanwhile, we tested inflammatory factor expression in major detoxification organs (liver and kidney). As is shown in Fig. 4E–J, the gene expression levels of TNF-α, IL-1α, IL-1β, IL-6, IL-10 and monocyte chemotactic protein-1 (MCP-1) in ASA6-NPs treated group were identical to the levels in control group, suggesting that ASA6-NPs probes do not lead to inflammatory responses in liver and kidney. In addition, no necrosis, congestion, inflammatory lesions and tissue damage was found in heart, kidney, liver, lung, spleen, bladder and brain in treated group at 7 days after intravenous injection of ASA6-NPs (Fig. 4K).

Next, in order to evaluate whether ASA6-NPs can be excreted via kidney, the biodistribution of ASA6-NPs in kidney was detected using chlorophosphonazo III, according to previous report [36]. From Fig. 4L, we can find that staining colors of ASA6-NPs treated group after 7 days and control group appear to be magenta, but the color of ASA6-NPs treated group at 1 day tend to be purple. As shown in Fig. 4L, red–green–blue (RGB) value analysis demonstrated the blue value of ASA6-NPs treated group after 1 day was much more than the value of control group and ASA6-NPs treated group after 7 days. Chlorophosphonazo III can react with Gd^3+^ component and become blue color, these results showed that there are almost no ASA6-NPs in the mouse kidney of ASA6-NPs treated group after 7 days. In addition, there was a peak of concentration of Gd^3+^ in urine at 6 h post-injection, and the concentration came down to near pre-injection level at 120 h post-injection (Additional file 1: Fig. S5). This result suggested that ASA6-NPs can be excreted in 7 days via kidney.

### Atherosclerosis targeted MR/NIR-II imaging with ASA6-NPs probe

**Fig. 5:**
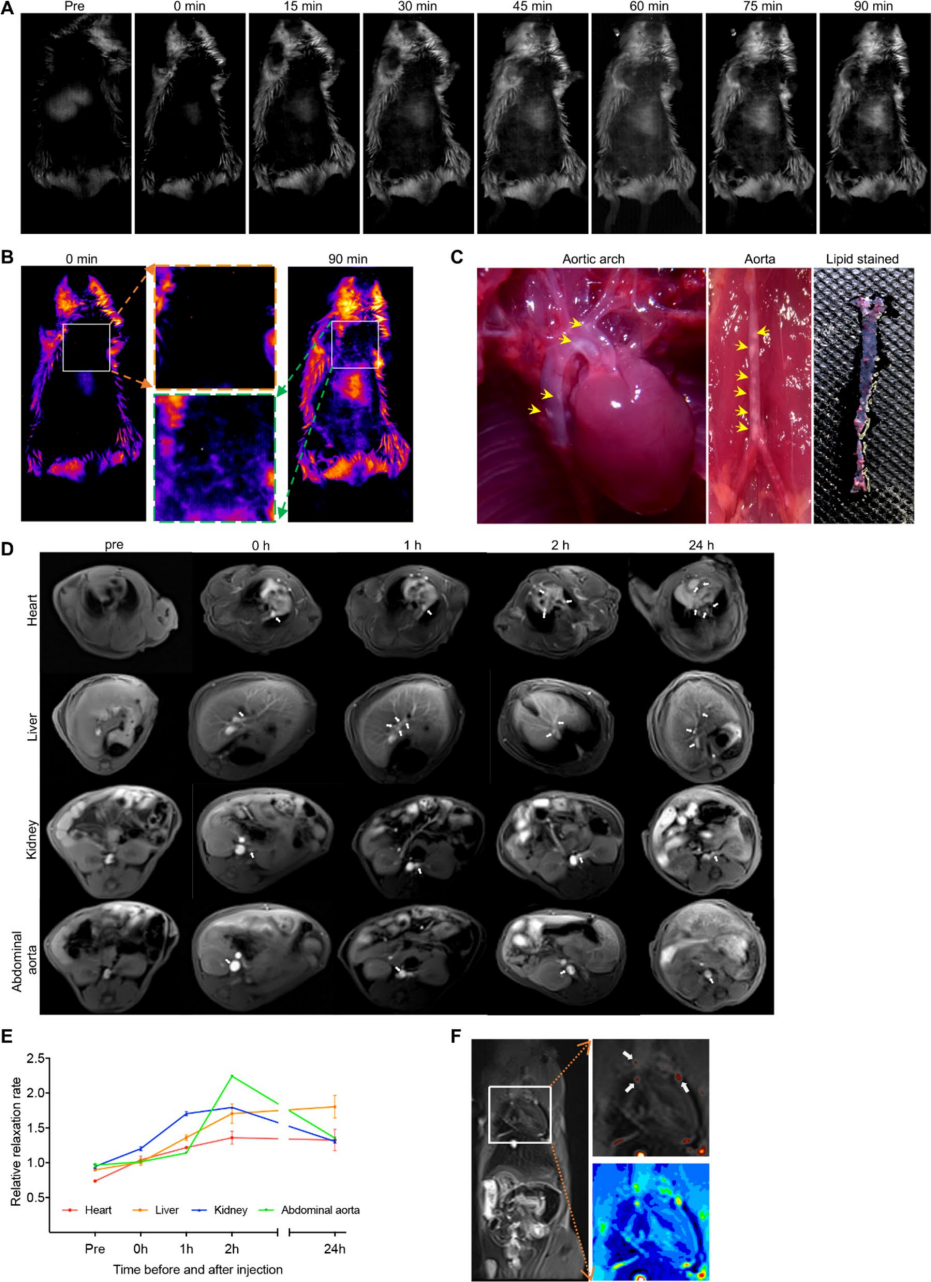
MR/NIR-II imaging of atherosclerotic plaque in atherosclerotic mice. **A** The NIR-II fluorescence imaging at different time points before and after the injection of ASA6-NPs probe in *ApoE*^−/−^ mice. **B** The pseudo-color image of NIR-II imaging at 0 and 90 min after ASA6-NPs probe injection. Close-up views of selected regions with positive signals were indicated. **C** The aortic arch and abdominal aorta in *ApoE*^−/−^ mice and lipid staining with Oil-red O. Yellow arrows indicate the atherosclerotic lesions. **D** The MRI of various mouse organs (including heart, liver, kidney and abdominal aorta) at different time points after ASA6-NPs injection. White arrows indicate the atherosclerotic lesions. **E** The dynamic changes of the MRI signals of various organs at different points. **F** The pseudo-color image of MRI signal in coronal position. White arrows indicate the atherosclerotic plaques. Data represent means ± s.d. from three independent replicates

For atherosclerotic imaging, an animal model with atherosclerotic plaques was established with *ApoE*^−/−^ mice which were fed a high-cholesterol diet. Upon intravenous injection of the ASA6-NPs probes, the NIR-II images were acquired at serial time points. Compared to pre-contrast image, ASA6-NPs probes accumulated in atherosclerotic plaques of aortic arch from 45 min after intravenous injection (Fig. 5A, B), and the increased fluorescence intensity at aortic arch was also observed during time span of 90 min. Then, the aortic arch and abdominal aorta of *ApoE*^−/−^ mice undergo NIR-II imaging were dissected, and the atherosclerotic plaque was observed, indicating the mice had remarkable atherosclerosis (Fig. 5C) which was consistent with NIR-II imaging result. In addition, the ASA6-NPs probe was also observed enriched in liver in the reason that liver is the primary metabolic center, and ASA6-NPs probe can also target to atherosclerotic plaques in liver artery (Fig. 5A, B). Meanwhile, distinctive fluorescence signal from abdominal aorta was also enhanced during NIR-II imaging (Fig. 5A, B).

We also perform targeted MR imaging for atherosclerosis using ASA6-NPs probes. Before and upon intravenous injection of ASA6-NPs probes, the MRI images were acquired in different organs at serial time points. As shown in Fig. 5D, MRI signal occurred immediately at heart, liver, kidney and abdominal aorta after ASA6-NPs probes injection, indicating atherosclerotic plaques were present in the artery. MRI images of the heart showed that several positive signals of atherosclerotic plaques derived from coronary artery. In detail, the results given in Fig. 5D showed that MRI signals of atherosclerotic plaques located in the right coronary artery (RCA) as well as left anterior descending (LAD), and signal in LAD was much stronger than RCA, indicating atherosclerosis in LAD was more serious than in RCA. As shown in liver image of Fig. 5D, positive signals of atherosclerotic plaques located in liver artery. The results given in kidney image of Fig. 5D showed signals of atherosclerotic plaques appeared in left renal artery. MRI signal peak appeared at 2 h after injection (Fig. 5E), indicating the accumulation of ASA6-NPs probes in atherosclerotic plaque, and the signal was still detectable at 24 h after injection (Fig. 5F). In order to evaluate the imaging specificity of ASA6-NPs, unconjugated NPs and non-atherosclerosis specific AFB1 scFv conjugated AFB1-NPs were intravenously injected into atherosclerosis model mice to perform MR imaging. As shown in Additional file 1: Fig. S6, the MRI signal in heart, liver and kidney of NPs group and AFB1-NPs group decreased significantly compared with ASA6-NPs group (Additional file 1: Fig. S6).

### Therapeutic effect of ASA6 antibody on atherosclerosis

**Fig. 6:**
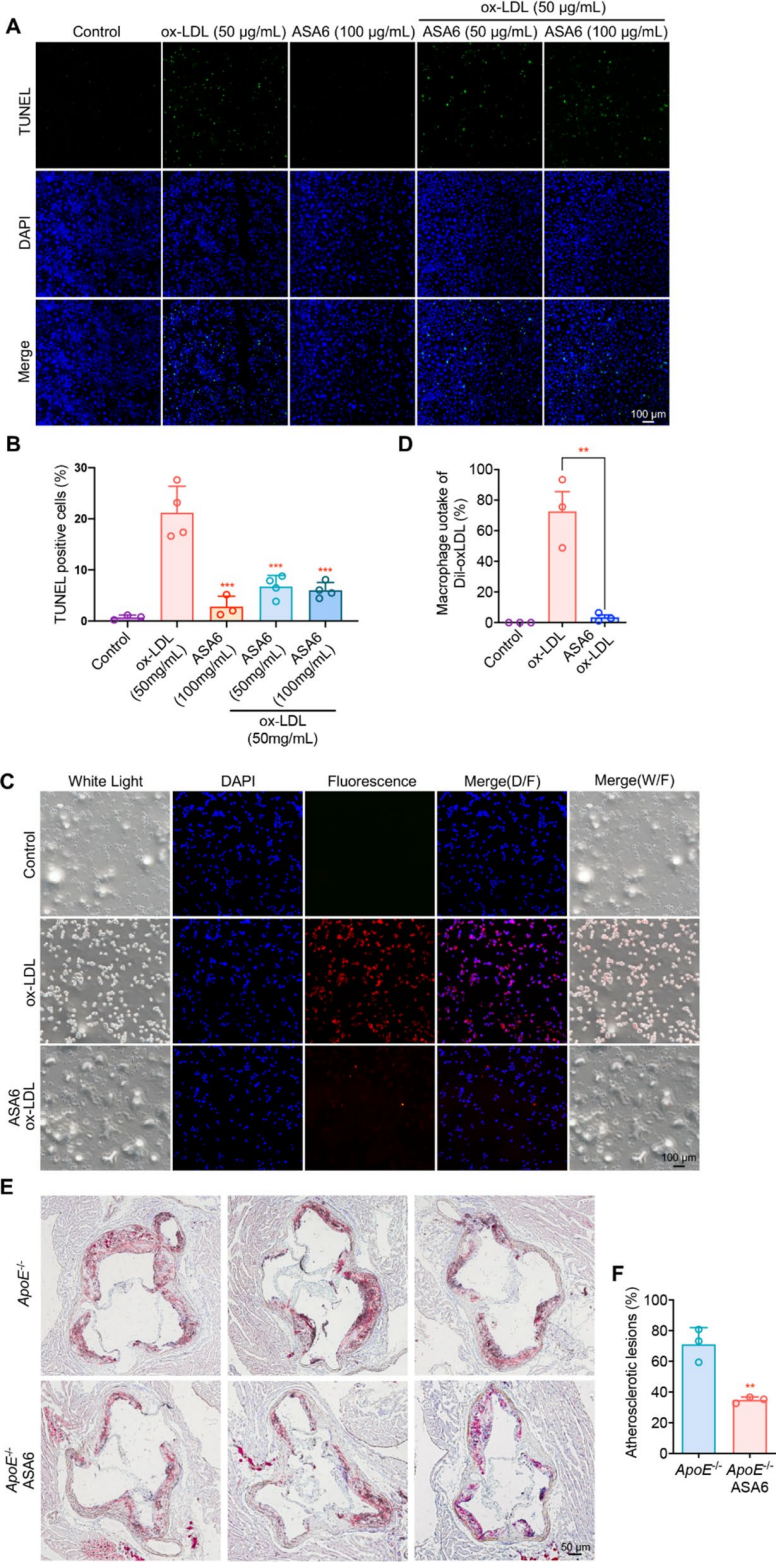
The therapeutic effects of ASA6 on atherosclerosis in vitro and in vivo. **A** Representative images of RAW 264.7 cell apoptosis detected by TUNEL staining. RAW 264.7 cells were treated with various concentrations of ASA6 and Ox-LDL. **B** The quantitative analysis of TUNEL positive cells. **C** The Dil-Ox-LDL uptake by RAW 264.7 cells. RAW 264.7 cells were treated with various concentrations of ASA6 and Ox-LDL. Blue fluorescence indicates cell nuclear. Red fluorescence indicates Dil-ox-LDL particles in RAW 264.7. **D** The quantitative analysis of the Dil-ox-LDL uptake. **E** Oil-red O staining of atherosclerotic lesions in aortic sinus in *ApoE*^−/−^ mice injected with PBS control or ASA6. **F** The quantitative analysis of atherosclerotic lesions. Data represent means ± s.d. from three independent replicates. ^**^p < 0.01, one-way ANOVA (**D**) or Student’s *t* test (**F**)

Previous study proved that Ox-LDL can induce cell apoptosis [41], so we assumed that ASA6 can inhibit Ox-LDL induced apoptosis by neutralizing Ox-LDL. TUNEL results in Fig. 6A, B showed that compared with Ox-LDL alone, ASA6 (50 and 100 µg/mL) can inhibit Ox-LDL induced RAW 264.7 macrophage cell apoptosis. To further investigate the protective effect of ASA6 to macrophage, we next performed the Dil-Ox-LDL uptake assay. As is shown in Fig. 6C, D, compared to Ox-LDL treated group, ASA6 can significantly inhibit the uptake of Ox-LDL by macrophages. The anti-atherosclerotic protective effect of ASA6 in vivo was also evaluated. As shown in Fig. 6E, F, the aortic root atherosclerotic lesion areas in ASA6 treated *ApoE*^−/−^ mice were decrease by almost 50% compared with *ApoE*^−/−^ control group. These results demonstrated ASA6 antibody can inhibit the uptake of Ox-LDL by macrophages, and attenuate Ox-LDL induced cell apoptosis to produce anti-atherosclerotic effect and decrease the lesion area.

### Therapeutic mechanisms of ASA6 antibody on atherosclerosis

**Fig. 7:**
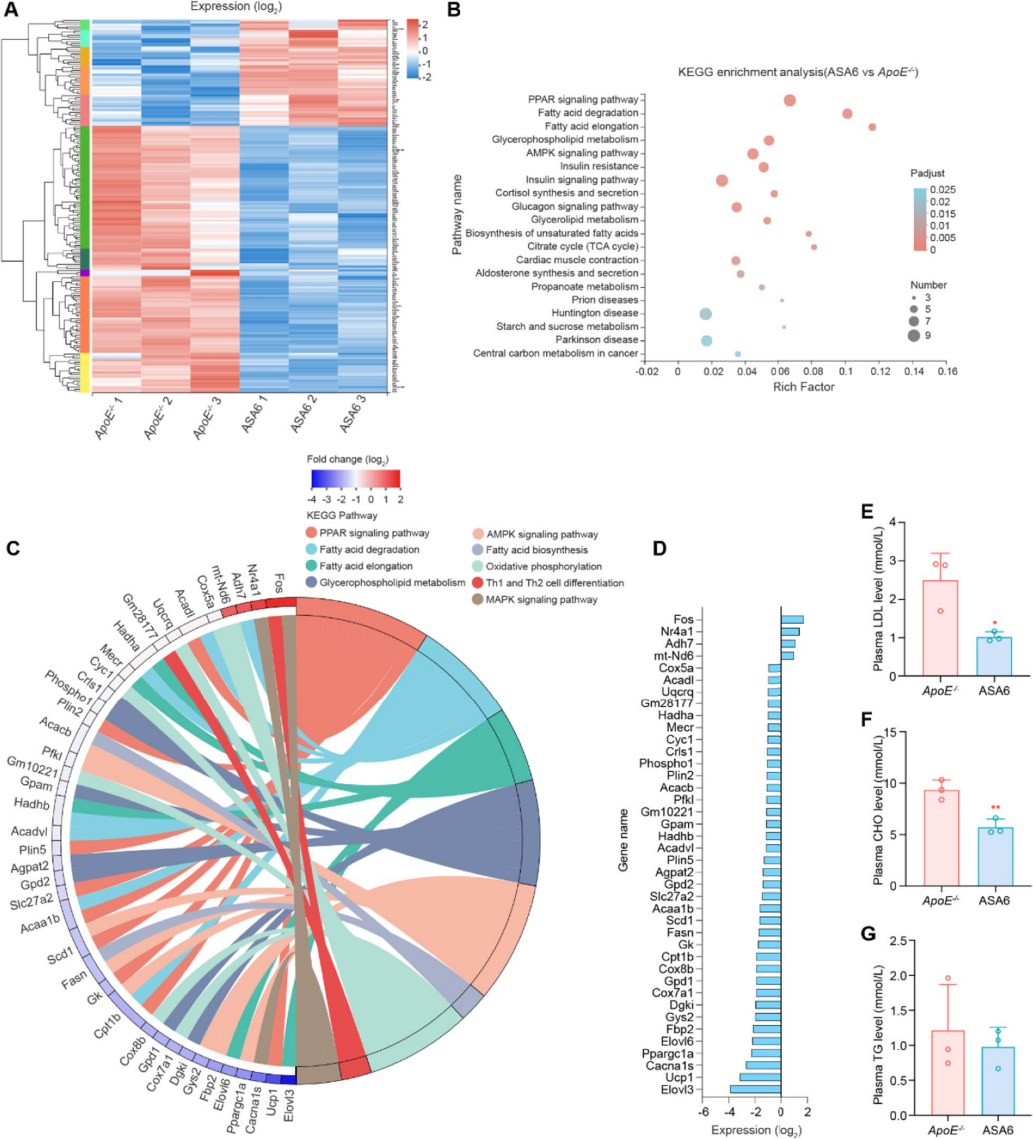
Therapeutic mechanisms of ASA6 on atherosclerosis. **A** Heat map of significantly up regulated and down regulated genes in aorta after ASA6 treatment (fold change ≥ 2 and *P* < 0.05). **B** KEGG pathway enrichment analysis of differentially expressed genes. The top 20 enriched pathways are shown. **C** Circos Plots of KEGG enrichment in lipid metabolism and macrophage polarization pathway related genes. **D** Relative expression levels of differentially expressed transcripts in aorta related to lipid metabolism and macrophage polarization pathway (data from** A**). **E**-**G** Plasma LDL, CHO and TG levels in *ApoE*^−/−^ control and ASA6 treated groups. Data represent means ± s.d. from three independent replicates. ^*^P < 0.05, ^**^P < 0.01, Student’s *t* test (**E**–**G**)

To further understand the therapeutic mechanism of ASA6 antibody, we performed transcriptome analysis on ASA6- and non-ASA6-treated *ApoE*^−/−^ mouse aorta. As shown in Additional file 1: Fig. S7A, the unguided principal component analysis (PCA) showed substantially different transcriptomic profiles between ASA6- and non-ASA6-treated *ApoE*^−/−^ mouse aorta. The heatmap of correlation between samples, shown in Fig. S7B, revealed that biological duplicates of each group were highly correlated. The Venn diagram shown in Additional file 1: Fig. S7C revealed that 13,764 genes were co-expressed in the two groups, while 374 genes were exclusively expressed in ASA6-treated group. The heatmap (Fig. 7A) and volcano plots in Additional file 1: Fig. S7D showed 179 significantly differentially expressed genes (DEGs), of which 128 genes were downregulated and 51 genes were upregulated.

Then we performed Kyoto Encyclopedia of Genes and Genomes (KEGG) pathway enrichment analysis based on DEGs. Interestingly, KEGG pathway enrichment analysis (Fig. 7B) indicated that PPAR signaling pathway, fatty acid degradation and elongation pathway, glycerophospholipid metabolism pathway and AMPK pathway were highly associated with the therapeutic mechanisms of ASA6 treatment. Nine significant KEGG terms are showed in chord plot (Fig. 7C), and the genes expression level related to the KEGG terms are shown in Fig. 7D. The genes related to fatty acid biosynthesis, including *Fasn* and *Acacb*, were downregulated in ASA6-treated group. Meanwhile, the down-regulated genes, such as *UCP1*, *Cpt1b*, *Gk*, *Scd1*, *Acaa1b*, *Scl27a2*, *Plin5*, *Plin2* and *Acadl*, may suggest that PPAR pathway was down-regulated in ASA6-treated mouse. Notably, some of these genes are also related to lipolysis, such as fatty acid degradation and oxidation. In addition, we noticed that the genes related to lipid oxidative phosphorylation (*Cox7a1*, *Cox8b*, *Cyc1*, *Uqcrq*, *Cox5a*) were also down-regulated in ASA6-treated group, which was consistent with the inactivation of PPAR pathway. Besides that, the genes related to fatty acid degradation and elongation (*Elovl3*, *Elovl6*, *Acaa1b*, *Acadvl*, *Hadhb*, *Merc*, *Hadha*, *Acadl*) were also down-regulated. Notably, genes related to Th1/Th2 cell differentiation and MAPK signaling pathway, including *Fos* and *Nr4a1*, were significantly up-regulated, suggested a shift of macrophage phenotype from a pro-inflammatory M1-like to a non-inflammatory M2-like after ASA6 antibody treatment. To analyze lipid metabolism, we perform plasma lipid analysis on ASA6- and non-ASA6-treated *ApoE*^−/−^ mouse, including LDL, CHO, and TG. As shown in Fig. 7E–G, the plasma LDL and CHO levels in the ASA6-treated group were significantly decreased compared with the control group, but still higher than the levels when fed with normal diet (Additional file 1: Fig. S8). Whereas, there was no significant change in the plasma TG, HDL, and Glucose levels between the two groups (Fig. 7G; Additional file 1: Fig. S9). GSEA analysis (Additional file 1: Fig. S10 A–C) revealed an enrichment of fatty acid biosynthesis and elongation pathway related genes in non-ASA6-treated *ApoE*^−/−^ control. To show the overview of changed metabolic pathways, we next performed interactive pathway (iPath) analysis (Additional file 1: Fig. S11), and the result showed the regulated metabolic pathways after ASA6 treatment mainly involved in lipid metabolism.

## Discussion

This study has demonstrated that human-derived ASA6-scFv can target to atherosclerotic plaques and inhibit atherosclerotic lesion formation in *ApoE*^*−/−*^ mice. Our data support that ASA6 antibody binds to oxidation-specific epitopes (OSEs) which are present on Ox-LDL and human atherosclerotic lesions. However, the exact structure of epitope to which ASA6 binds is not fully characterized until now.

There is overwhelming evidence that Ox-LDL is hallmarks of high cardiovascular disease risk and prevalent within atherosclerotic plaques [42]. Native LDL penetrates the intima and is modified into Ox-LDL by oxidative stress during atherogenesis. Ox-LDL is immunogenic and can be recognized by pattern recognition receptors (PPRs) of innate immunity, such as scavenger receptors (SRs) [43]. The recognition and uptake of Ox-LDL by SRs (e.g. CD36, SRA1, SRA2, SRB1 and LOX1) leads to cholesterol crystals formation and chronic inflammation. The hypothesis that the inhibition of SRs mediated uptake of Ox-LDL with oxidation-specific antibody could treat atherosclerosis in animal model has been proved [23, 44]. Our results showed ASA6-scFv could inhibit the uptake of Ox-LDL by macrophages and attenuate the macrophage apoptosis induced by Ox-LDL. The inhibition of ASA6 to Ox-LDL was achieved by binding to OSEs on Ox-LDL other than by interfering with macrophage SRs function. This result support the hypothesis that therapies by binding and inactivating Ox-LDL with specific antibodies may be beneficial for treating atherosclerosis.

Due to the complexity and heterogeneity of Ox-LDL, B cells can react with various autoantigens derived from Ox-LDL by producing either IgM or IgG antibodies [2]. In general, these autoantibodies to Ox-LDL can be found in patients with coronary artery disease [13, 14]. The single-chain antibody phage library in this study was made from rearranged *V*_*H*_ and *V*_*L*_ gene pools from 50 patients undergoing clinical indicated coronary angiography. The oxidation-specific ASA6-scFv isolated from this phage library is a linear protein in which the antigen-binding domains (V_H_ and V_L_) are joined by a flexible peptide linker that allows appropriate folding of the expressed protein. The ASA6-scFv lacks the Fc fragment of intact antibodies, so the anti-atherosclerosis effect was caused solely by binding to oxidation-specific epitopes and inhibit Ox-LDL uptake by macrophages. The ASA6 antibody fragment also has the potential to be engineered into full-length antibody or scFv-Fc fusion, which can improve pharmacokinetics and effector function. More importantly, given ASA6-scFv is derived from human antibody library and the immunogenicity is very low, it will be theoretically safer for clinical translation with no need of structural optimization for human use.

Transcriptome analysis of mouse aorta was performed to reveal the mechanism of atherosclerosis therapy by ASA6. Interestingly, the transcriptomic changes of ASA6-treated group were observed mainly involved in lipid metabolism-related pathways, such as PPAR, lipolysis and lipogenesis pathways. Among these pathways, lipolysis related genes (e.g., *UCP1*, *Cpt1b*, *Cox7a1* and *Cox8b*) are crucial enzymes for oxidative and lipid degradation, and are observed down-regulated after ASA6 treatment. This result is consistent with previous findings that uncoupling protein 1 (UCP1)-dependent lipolysis could promote atherosclerotic plaque growth and instability [45]. The genes related to lipid metabolism pathway, including cholesterol metabolism and PPAR signaling pathways were highly expressed in foamy macrophages [46], and PPAR pathway was found to improve lipid accumulation in immune cells [47]. The repression of PPAR pathway related genes was also proved to play a protective role in atherosclerosis [48]. So, the down-regulation of PPAR pathway in our study indicated that the formation of foam cells was suppressed after ASA6 treatment.

We also found that genes involved in fatty acid biosynthesis, such as *Slc27a2*, *Acacb*, *Elovl3* and *Elovl6*, were down-regulated in ASA6-treated mouse. Interestingly, ASA6 can also reduce the fatty acid transport proteins (FATP) gene expression, indicating that the free fatty acid (FFA) transport from the extracellular into the intracellular may be inhibited. The FATP protein family, which are classified as members of solute carrier 27 (Slc27), are the key transporter and enzyme of fatty acid, especially long chain fatty acids (LCFAs) [49, 50]. Moreover, ASA6 can inhibit the gene expression of acetyl-Coenzyme A carboxylase beta (*Acacb*) and reduce the production of malonyl-CoA, and is supposed to inhibit endogenous fatty acid synthesis. Besides, long-chain fatty acid elongase (e.g., *Elovl3* and *Evlov6*) play very important roles in the elongation of very long chain-saturated fatty acids (VLC-SFA) [51]. We speculate ASA6 can reduce VLS-SFA levels through reducing *Elovl3 and Evlov6* gene expressions. Consistent with transcriptomic analysis, both the plasma LDL level and the plasma total cholesterol (CHO) level in ASA6-treated group were lower than that in *ApoE*^*−*/*−*^ group. Taken together, ASA6 can decrease the macrophage intracellular lipid accumulation by directly blocking Ox-LDL uptake and decreasing plasma LDL and CHO level. Consistent with this speculation, oil red O staining results showed ASA6 could decrease the amount and size of atherosclerotic lipid lesion in aorta.

The changes in intracellular metabolic pathways can alter the function of highly plastic cells, such as macrophages, which is known as immunometabolism [52, 53]. Our results showed ASA6 single-chain antibody could regulate lipid metabolism pathway, we assume this could also influence the function of macrophages. In atherosclerosis, the heterogeneity of macrophages is simplified as M1/M2 classification [54]. The M1 macrophages are driven by INFγ, LPS, Ox-LDL and cholesterol crystals, and display a pro-inflammatory profile associated with plaque instability. The M2 macrophages are induced by IL-4 which are anti-inflammatory and prevent foam cell formation. Nuclear receptor NR4A1 (Nur77) was reported to be indispensable for monocyte differentiation into M2 macrophage, and the deletion of NR4A1 could increase atherosclerosis [55, 56]. Transcription factor c-Fos was also found to play an important role in regulation of macrophages differentiation into M2 phenotype [56]. Our study showed the genes expression of NR4A1 and c-Fos were up-regulated after ASA6 treatment. This result indicates ASA6 can facilitate a shift of macrophage phenotype from inflammatory M1 to attenuated M2, which is consistent with the result of another oxidized phospholipids (OxPL) specific antibody therapy study [44]. Taken together, ASA6 can regulate lipid metabolism further to produce “lipid blockade” effect, and this phenomenon is rarely reported. Therefore, we will study the functions and mechanisms of the “lipid blockade” effect in atherosclerosis in the future, by analyzing metabolism and gene expression in foam cells, and to illustrate more definite mechanisms of ASA6 therapeutic effects.

Animal and clinical studies demonstrated that Ox-LDL was enriched in vulnerable atherosclerotic plaques [57, 58], and the prevalent of OSEs were closely related to plaque disruption and acute coronary syndromes in patients [59, 60]. The specific uptake of ASA6 by human atherosclerotic plaques was proved in this study. Thus, OSE targeted ASA6-NPs probe might allow for the identification and quantification of atherosclerotic lesions and evaluation of plaque vulnerability. The NaNdF_4_ inner core and NaGdF_4_ outer shell endow ASA6-NPs with NIR-II fluorescence and MR properties with low autofluorescence background, minimal scattering and unlimited penetration depth for in vivo dual-modal imaging. The hydrated mean diameter of ASA6-NPs was 14.42 nm which was much smaller than mouse aortic endothelial gap junctions (approximately 147 nm to 437 nm) [61]. Small size of ASA6-NPs facilitated the uptake and diffusion into arterial wall, and also help the excretion out of the body. Maximum MRI signal enhancement in this study was observed at 2 h post-injection, which was earlier than previous reports using oxidation-specific antibody conjugated Gd or Mn contrast agent (48–72 h post-injection) [26, 62], suggesting a fast uptake of ASA6-NPs probe by atherosclerotic lesions. In this study, ASA6-NPs probe can be excreted through kidney within 7 days after injection. With the help of high imaging quality, sensitivity and resolution of MRI imaging, the oxidation-specific epitope was also observed in liver and kidney in our study, which will be potentially beneficial for the diagnosis of steatosis disease in these organs. The advantages of fluorescence-based optical image technique are high temporal resolution and fast imaging capability [63]. In our study, NIR-II signal was detectable as early as 45 min after injection of ASA6-NPs probe. The in vitro and in vivo biocompatibility of ASA6-NPs and success of atherosclerotic imaging in this study demonstrates the promise of antibody based molecular probe for clinical translation. We will focus on investigating the long-term toxicity of ASA6-NPs and eventually translating into clinical applications in the future.

## Conclusions

In summary, we demonstrated that human oxidation-specific scFv antibody ASA6 can inhibit the uptake of Ox-LDL by macrophages, and reduce the macrophage apoptosis induced by Ox-LDL. When treated *ApoE*^*−/−*^ mouse with ASA6, the progression of atherosclerotic lesions was inhibited. Interestingly, when injected in mouse model, ASA6 can regulate the lipid metabolism and macrophage polarization pathway related genes expression, which may contribute to the anti-atherosclerotic effect. ASA6 antibody has been shown strongly binding to human aortic plaques, and can be used in atherosclerosis MR/NIR-II imaging when conjugated with nanoparticles. Our results have solidly demonstrated the potential of ASA6 antibody in clinical use for therapy and imaging of atherosclerosis.

## Supplementary information

**Additional file 1: Figure S1.** Phage ELISA of selected individual clones towards human atherosclerotic plaque. **Figure S2**. The absorption spectra of NaNdF_4_@NaGdF_4_ nanoparticles. **Figure S3**. The NIR-II fluorescence spectrum of NaNdF_4_@NaGdF_4_under 808 nm excitation. **Figure S4**. In vitro longitudinal relativity against concentration gradient of RE^3+^ (Nd^3+ ^+ Gd3^+^) ions of NaNdF_4_@NaGdF_4_. **Figure S5**. Concentration of Gd in urine at different time points. **Figure S6**. MR imaging of atherosclerotic plaque using NaNdF_4_@NaGdF_4_ and AFB1-NaNdF_4_@NaGdF_4_. **Figure S7**. Mice aorta transcriptome analysis after ASA6 treatment. **Figure S8**. Plasma LDL, CHO and TG levels in ApoE^–/–^ mice when fed with normal diet. **Figure S9**. Plasma glucose (GLU) and high-density lipoprotein (HDL) levels in ApoE^–/–^ control and ASA6 treated groups. **Figure S10**. Gene set enrichment analysis of lipid metabolism between ASA6-treated group and control group. **Figure S11**. iPath analysis of the differently expressed genes (DEGs) after ASA6 treatment. **Table S1**. Clinical characteristics of CAD patients who donated blood samples. **Table S2**. Sequences of the primers used for scFv phage display library construction. **Table S3**. Enrichment of specific recombinant phages to human atherosclerosis during panning cycles. **Table S4**. Sequences of the primers used for inflammatory cytokines qRT-PCR.

## Data Availability

All data associated with this study are present in the paper or Supplementary Materials.
