# Supplementary material for: An atherosclerotic plaque-targeted single-chain antibody for MR/NIR-II imaging of atherosclerosis and anti-atherosclerosis therapy
Source: J Nanobiotechnology. 2021 Sep 28;19:296. doi: 10.1186/s12951-021-01047-4 (PMC8479957; doi:10.1186/s12951-021-01047-4)
Supplement: Supplementary file 1 — Additional file 1: Figure S1. Phage ELISA of selected individual clones towards human atherosclerotic plaque. Figure S2. The absorption spectra of NaNdF4@NaGdF4 nanoparticles. Figure S3. The NIR-II fluorescence spectrum of NaNdF4@NaGdF4under 808 nm excitation. Figure S4. In vitro longitudinal relativity against concentration gradient of RE3+ (Nd3+ + Gd3+) ions of NaNdF4@NaGdF4. Figure S5. Concentration of Gd in urine at different time points. Figure S6. MR imaging of atherosclerotic plaque using NaNdF4@NaGdF4 and AFB1-NaNdF4@NaGdF4. Figure S7. Mice aorta transcriptome analysis after ASA6 treatment. Figure S8. Plasma LDL, CHO and TG levels in ApoE–/– mice when fed with normal diet. Figure S9. Plasma glucose (GLU) and high-density lipoprotein (HDL) levels in ApoE–/– control and ASA6 treated groups. Figure S10. Gene set enrichment analysis of lipid metabolism between ASA6-treated group and control group. Figure S11. iPath analysis of the differently expressed genes (DEGs) after ASA6 treatment. Table S1. Clinical characteristics of CAD patients who donated blood samples. Table S2. Sequences of the primers used for scFv phage display library construction. Table S3. Enrichment of specific recombinant phages to human atherosclerosis during panning cycles. Table S4. Sequences of the primers used for inflammatory cytokines qRT-PCR. [file 12951_2021_1047_MOESM1_ESM.docx]

**Additional file 1**

**An atherosclerotic plaque-targeted single-chain antibody for MR/NIR-II imaging of atherosclerosis and anti-atherosclerosis therapy**

Liwei Zhang^1†^, Sheng Xue^1†*^, Feng Ren^2†^, Siyang Huang^1^, Ruizhi Zhou^3^, Yu Wang^1^, Changyong Zhou^1^, Zhen Li^2*^

^1^Institute for Translational Medicine, The Affiliated Hospital of Qingdao University, College of Medicine, Qingdao University, Qingdao, 266021 China.

^2^Center for Molecular Imaging and Nuclear Medicine, State Key Laboratory of Radiation Medicine and Protection, School for Radiological and Interdisciplinary Sciences (RAD-X), Soochow University, Collaborative Innovation Center of Radiation Medicine of Jiangsu Higher Education Institutions, Suzhou 215123, P. R. China.

^3^Department of Radiology, The Affiliated Hospital of Qingdao University, Qingdao University, Qingdao, 266021 China.

^†^L.Z., S.X., and F.R. contributed equally to this work.

^*^Corresponding author: shengxue@qdu.edu.cn; zhenli@suda.edu.cn

**
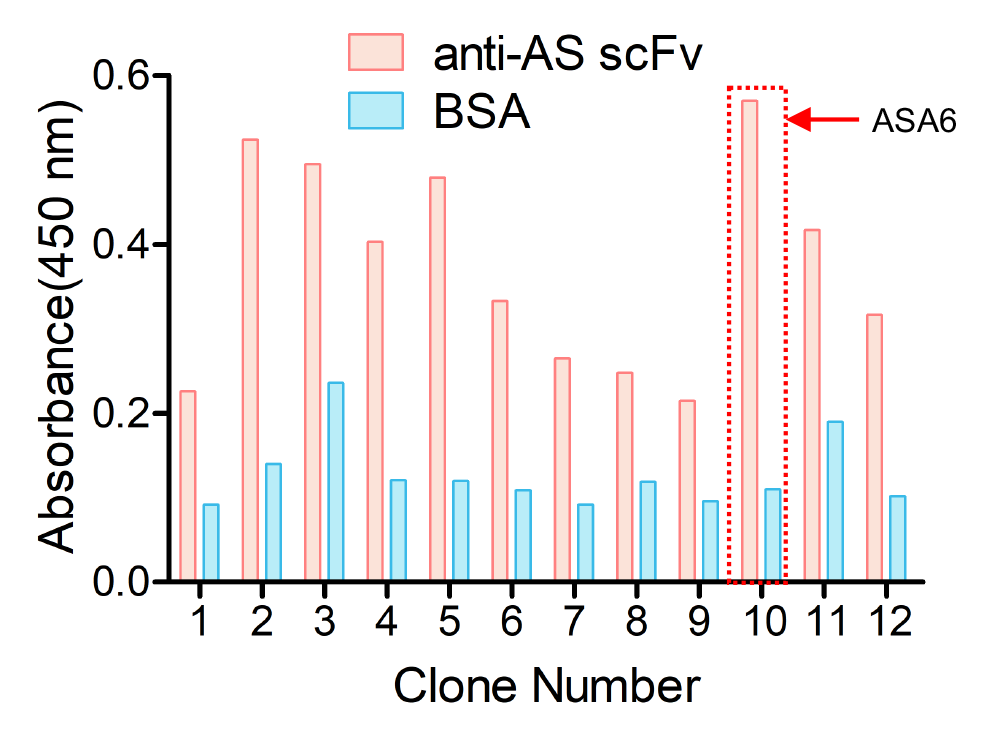
**

**Figure S1.** **Phage ELISA of selected individual clones towards human atherosclerotic plaque.** Twelve phage clones were confirmed high affinity for atherosclerotic plaques compared with BSA by phage ELISA. Red box represents one phage clone, named as ASA6, showing the strongest affinity.





**Figure S2. The absorption spectra of NaNdF_4_@NaGdF_4_ nanoparticles.**





**Figure S3. The NIR-II fluorescence spectrum of NaNdF_4_@NaGdF_4_ under 808 nm excitation.**





**Figure S4. In vitro longitudinal relativity against concentration gradient of RE^3+^ (Nd^3+^ + Gd3^+^) ions of NaNdF_4_@NaGdF_4_.**


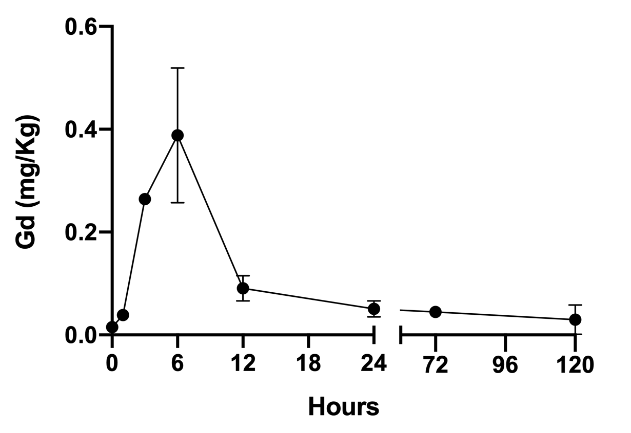


**Figure S5. Concentration of Gd in urine at different time points.**


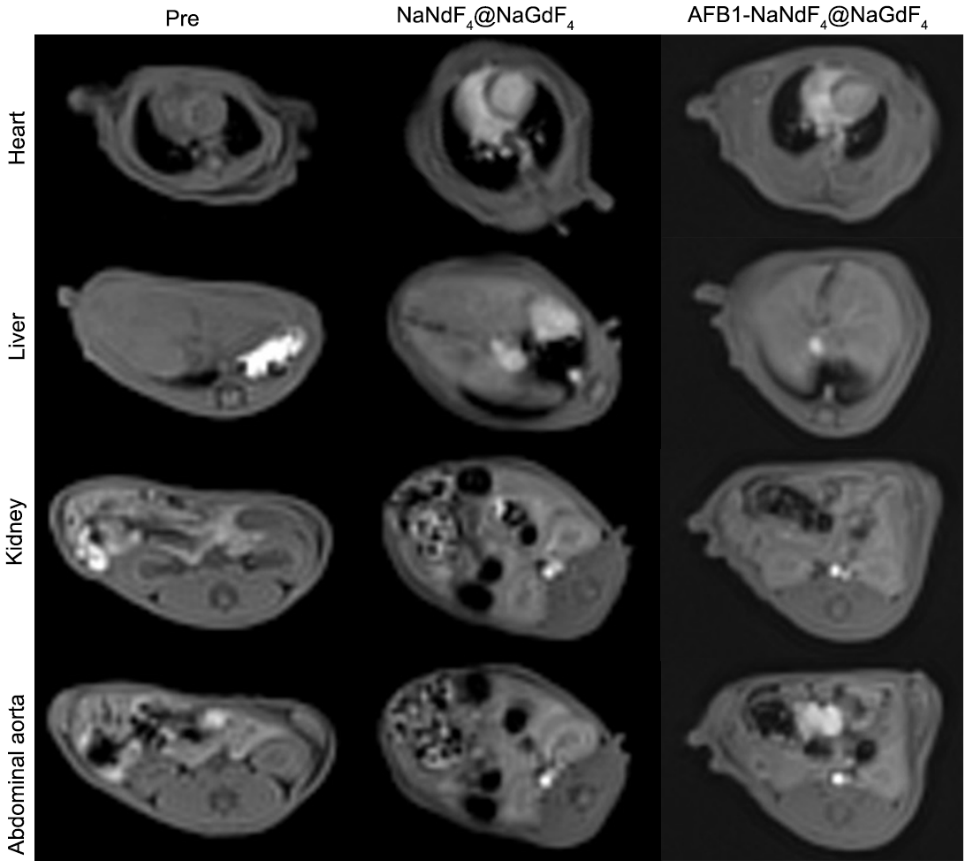


**Figure S6. MR imaging of atherosclerotic plaque using NaNdF_4_@NaGdF_4_ and AFB1-NaNdF_4_@NaGdF_4_.** NaNdF_4_@NaGdF_4_ and AFB1-NaNdF_4_@NaGdF_4_ probe (Gd concentration of 1.39 mg/mL) was intravenously injected (100 μL per mouse), and MR imaging was performed immediately post-injection.


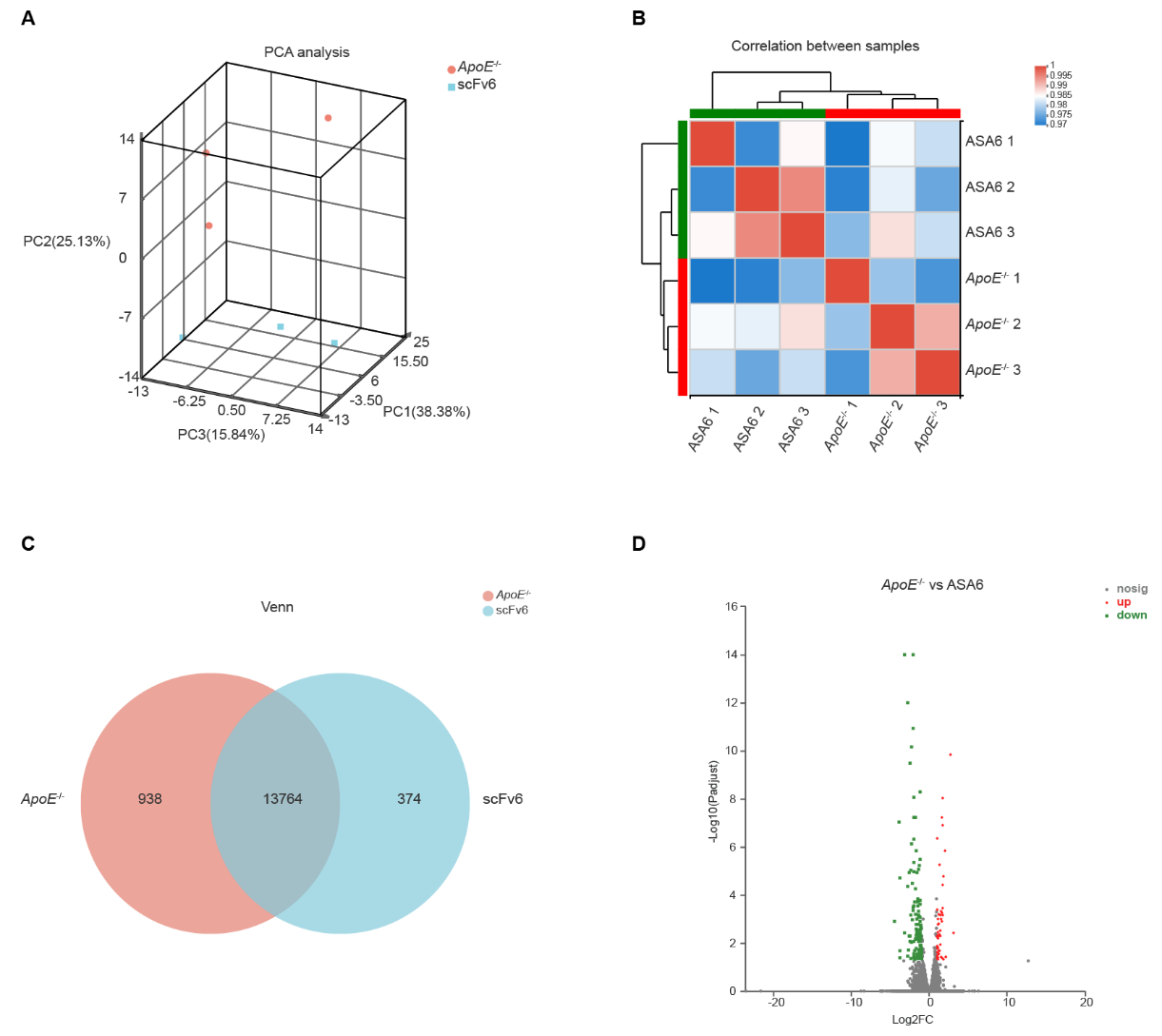


**Figure S7. Mice aorta transcriptome analysis after ASA6 treatment.** (A) Principal component analysis (PCA) was performed based on differentially expressed genes from aorta of different groups. PCA analysis of each sample is indicated by data point. (B) Heatmap of correlation between samples of two groups. (C) Venn diagram of the transcriptomic profiles between *ApoE*^−/−^ and ASA6 group. (D) Volcano plots showing the identified upregulated and downregulated genes between two groups.


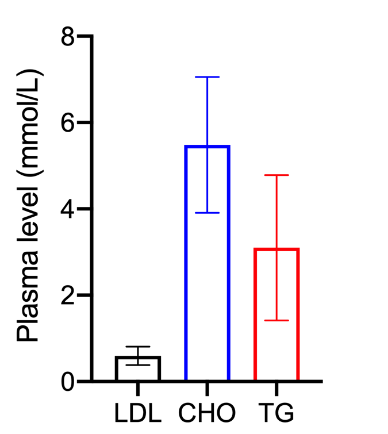


**Figure S8. Plasma LDL, CHO and TG levels in ApoE^–/–^ mice when fed with normal diet.**


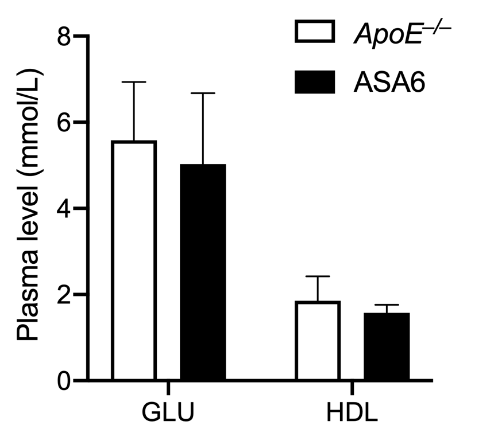


**Figure S9. Plasma glucose (GLU) and high-density lipoprotein (HDL) levels in ApoE^–/–^ control and ASA6 treated groups.**


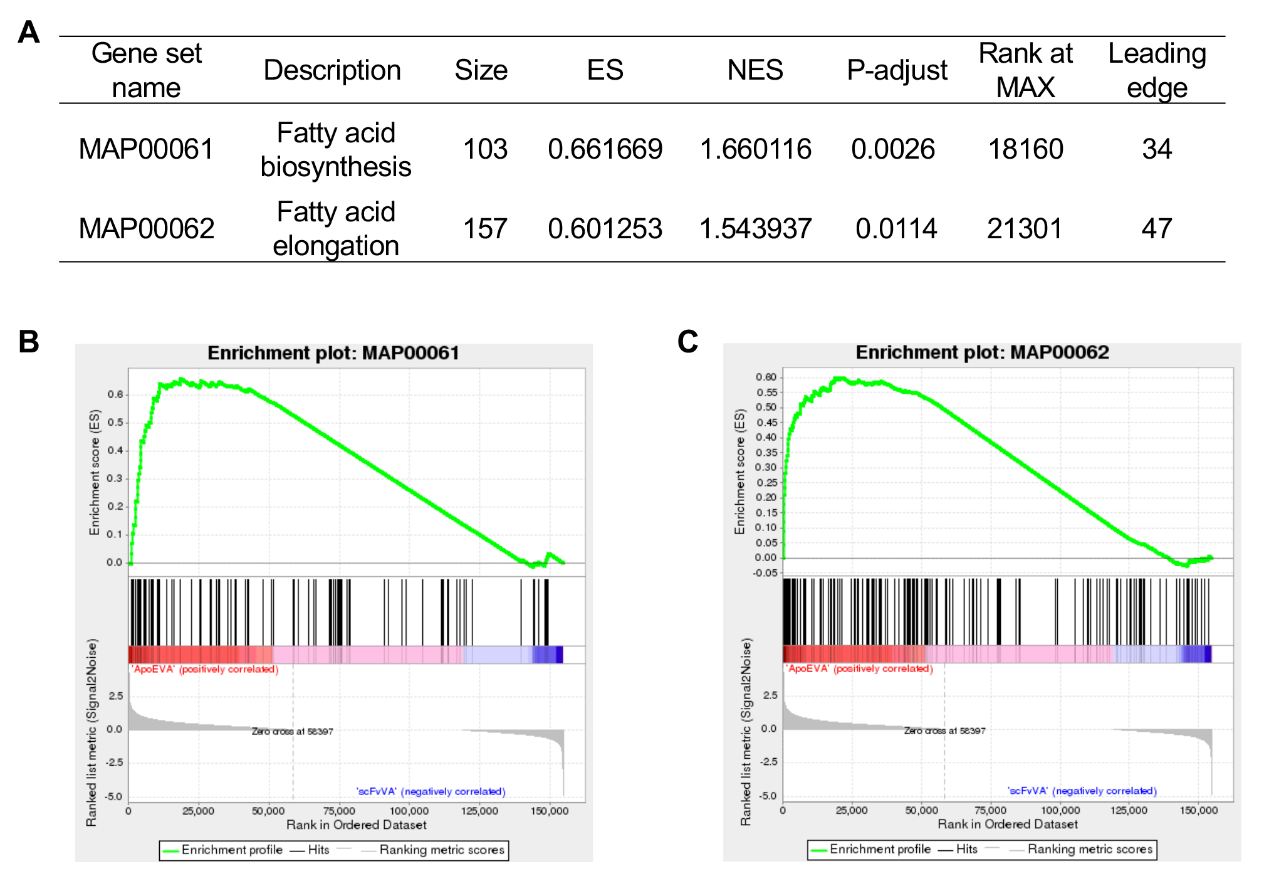


**Figure S10.** **Gene set enrichment analysis of lipid metabolism between ASA6-treated group and control group.** (A) The information of different gene sets between the two groups. ES: enrichment score. NES: normalized enrichment score. The *P*-adjust in the graphs were calculated by GSEA analysis. (B) The represent GSEA of fatty acid biosynthesis. (C) The represent GSEA of fatty acid elongation.


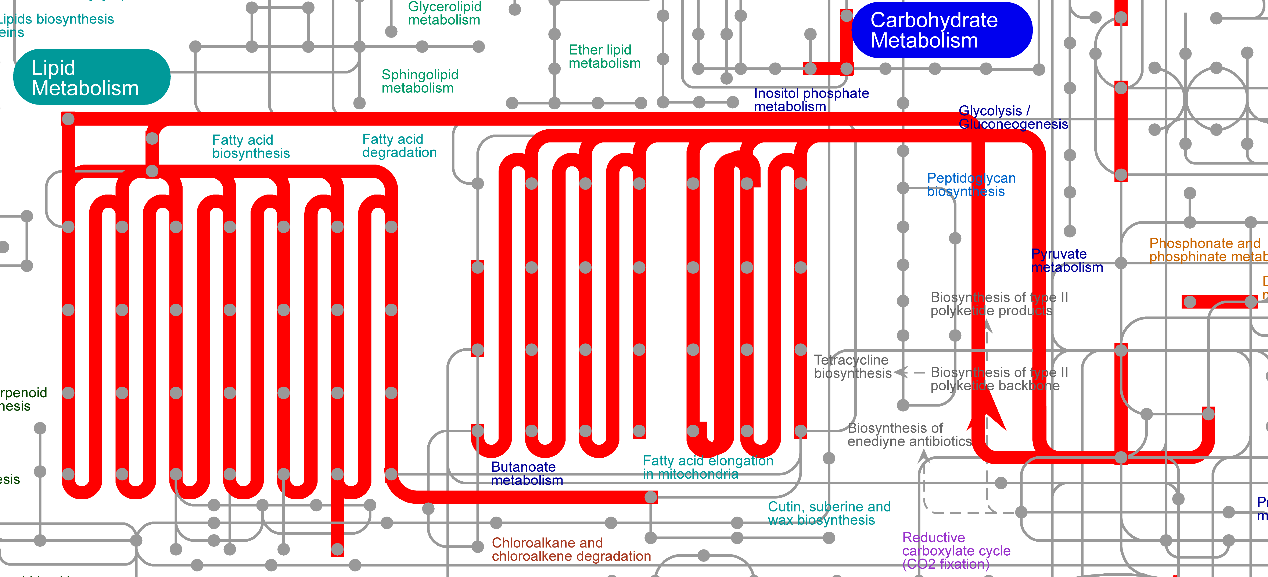


**Figure S11. iPath analysis of the differently expressed genes (DEGs) after ASA6 treatment.** DEGs were mapped onto the lipid metabolism map. The red lines indicate upregulated metabolic pathways (*ApoE*^−/−^ group vs ASA6 group).

**Supplementary Table 1. Clinical characteristics of CAD patients who donated blood samples.** BMI, Body mass index; DM, Diabetes mellitus; SBP, systolic blood pressure; DBP, diastolic blood pressure; TC, total cholesterol; TG, total triglycerid; HDL, high-density lipoprotein; LDL, low-density lipoprotein. Data are shown as the mean ± SD.

| Variable |  | | | |
| --- | --- | --- | --- | --- |
| Male/Female (n/n) |  |  | 43/12 |  |
| Age (years) |  | | 65.6±12.4 | |
| Weight (kg) |  | | 68.5±15.7 | |
| BMI (kg/m^2^) |  | | 24.1±5.0 | |
| Heart Rate (beats/minutes) |  | | 73.6±15.1 | |
| SBP (mmHg) |  | | 124.4±18.5 | |
| DBP (mmHg) |  | | 76.2±10.5 | |
| NYHA Functional Classification | I | | 34（61.8%） | |
|  | II | | 18（32.8%） | |
|  | III | | 2（3.6%） | |
|  | IV | | 1（1.8%） | |
| Smoking (%) |  | | 25（45.6%） | |
| Alcohol drinking (%) |  | | 20（36.4%） | |
| Hypertension (%) |  | | 34（61.8%） | |
| Diabetes (%) |  | | 20（36.4%） | |
| Fasting Blood Glucose (mM) |  | | 6.5±3.0 | |
| Glycated Albumin (%) |  | | 13.8±4.9 | |
| CHO (mM) |  | | 4.7±1.1 | |
| TG (mM) |  | | 1.7±1.1 | |
| ApoB (g/L) |  | | 1.0±0.2 | |
| HDL (mM) |  | | 1.1±0.3 | |
| LDL (mM) |  | | 2.9±0.8 | |
| Lipoprotein (a) (mg/L) |  | | 281.3±253.0 | |
| Statin Drug (%) |  | | 54（98.2%） | |

**Supplementary Table 2. Sequences of the primers used for scFv phage display library construction**

| Primer name | Sequence |
| --- | --- |
| HSCVH1-FL | GGTGGTTCCTCTAGATCTTCCTCCTCTGGTGGCGGTGGCTCGGGCGGTGGTGGGCAGGTGCAGCTGGTGCAGTCTGG |
| HSCVH2-FL | GGTGGTTCCTCTAGATCTTCCTCCTCTGGTGGCGGTGGCTCGGGCGGTGGTGGGCAGATCACCTTGAAGGAGTCTGG |
| HSCVH35-FL | GGTGGTTCCTCTAGATCTTCCTCCTCTGGTGGCGGTGGCTCGGGCGGTGGTGGGGAGGTGCAGCTGGTGSAGTCTGG |
| HSCVH3a-FL | GGTGGTTCCTCTAGATCTTCCTCCTCTGGTGGCGGTGGCTCGGGCGGTGGTGGGGAGGTGCAGCTGKTGGAGTCTG |
| HSCVH4-FL | GGTGGTTCCTCTAGATCTTCCTCCTCTGGTGGCGGTGGCTCGGGCGGTGGTGGGCAGGTGCAGCTGCAGGAGTCGGG |
| HSCVH4a-FL | GGTGGTTCCTCTAGATCTTCCTCCTCTGGTGGCGGTGGCTCGGGCGGTGGTGGGCAGGTGCAGCTACAGCAGTGGGG |
| HSCG 1234-B | CCTGGCCGGCCTGGCCACTAGTGACCGATGGGCCCTTGGTGGARGC |
| HSCM-B | CCTGGCCGGCCTGGCCACTAGTAAGGGTTGGGGCGGATGCACTCCC |
| HSCA-B | CCTGGCCGGCCTGGCCACTAGTGACCTTGGGGCTGGTCGGGGATGC |
| HSCD-B | CCTGGCCGGCCTGGCCACTAGTCACATCCGGAGCCTTGGTGGGTGC |
| HSCE-B | CCTGGCCGGCCTGGCCACTAGTGACGGATGGGCTCTGTGTGGAGGC |
| HSCKl-F | GGGCCCAGGCGGCCGAGCTCCAGATGACCCAGTCTCC |
| HSCK24-F | GGGCCCAGGCGGCCGAGCTCGTGATGACYCAGTCTCC |
| HSCK3-F | GGGCCCAGGCGGCCGAGCTCGTGWTGACRCAGTCTCC |
| HSCK5-F | GGGCCCAGGCGGCCGAGCTCACACTCACGCAGTCTCC |
| HSCJK14o-B | GGAAGATCTAGAGGAACCACCTTTGATYTCCACCTTGGTCCC |
| HSCJK2o-B | GGAAGATCTAGAGGAACCACCTTTGATCTCCAGCTTGGTCCC |
| HSCJK3o-B | GGAAGATCTAGAGGAACCACCTTTGATATCCACTTTGGTCCC |
| HSCJK5o-B | GGAAGATCTAGAGGAACCACCTTTAATCTCCAGTCGTGTCCC |
| HSCLam1a | GGGCCCAGGCGGCCGAGCTCGTGBTGACGCAGCCGCCCTC |
| HSCLamlb | GGGCCCAGGCGGCCGAGCTCGTGCTGACTCAGCCACCCTC |
| HSCLam2 | GGGCCCAGGCGGCCGAGCTCGCCCTGACTCAGCCTCCCTCCGT |
| HSCLam3 | GGGCCCAGGCGGCCGAGCTCGAGCTGACTCAGCCACCCTCAGTGTC |
| HSCLam4 | GGGCCCAGGCGGCCGAGCTCGTGCTGACTCAATCGCCCTC |
| HSCLam6 | GGGCCCAGGCGGCCGAGCTCATGCTGACTCAGCCCCACTC |
| HSCLam78 | GGGCCCAGGCGGCCGAGCTCGTGGTGACYCAGGAGCCMTC |
| HSCLam9 | GGGCCCAGGCGGCCGAGCTCGTGCTGACTCAGCCACCTTC |
| HSCLam l0 | GGGCCCAGGCGGCCGAGCTCGGGCAGACTCAGCAGCTCTC |
| HSCJLam 1236 | GGAAGATCTAGAGGAACCACCGCCTAGGACGGTCASCTTGGTSCC |
| HSCJLam4 | GGAAGATCTAGAGGAACCACCGCCTAAAATGATCAGCTGGGTTCC |
| HSCJLam57 | GGAAGATCTAGAGGAACCACCGCCGAGGACGGTCAGCTSGGTSCC |
| RSC-F | GAGGAGGAGGAGGAGGAGGCGGGGCCCAGGCGGCCGAGCTC |
| RSC-B | GAGGAGGAGGAGGAGGAGCCTGGCCGGCCTGGCCACTAGTG |

**Supplementary Table 3. Enrichment of specific recombinant phages to human atherosclerosis during panning cycles**

| Round | Input Phage  (PFU mL^-1^) ^a^ | Output Phage  (PFU mL^-1^) | Phage Recovery |
| --- | --- | --- | --- |
| 1 | 2.9 × 10^12^ | 4.0 × 10^4^ | 1.4 × 10^−8^ |
| 2 | 4.4 × 10^12^ | 6.4 × 10^4^ | 1.5 × 10^−8^ |
| 3 | 5.0 × 10^11^ | 1.6 × 10^6^ | 3.2 × 10^−6^ |

^a^ PFU, plaque-forming unit

**Supplementary Table 4. Sequences of the primers used for inflammatory cytokines qRT-PCR**

| Gene name | Forward primer | Reverse primer |
| --- | --- | --- |
| Mouse GAPDH | 5’-AGGTCGGTGTGAACGGATTTG-3’ | 5’-TGTAGACCATGTAGTTGAGGTCA-3’ |
| Mouse TNF-α | 5’-ACAAGGCTGCCCCGACTAC-3’ | 5’-TGGGCTCATACCAGGGTTTG-3’ |
| Mouse IL-1α | 5’-GATGAAGCTCGTCAGGCAGAA-3’ | 5’-CCTCCCGACGAGTAGGCATA-3’ |
| Mouse IL-1β | 5’-CTTTCCCGTGGACCTTCCA-3’ | 5’-CTCGGAGCCTGTAGTGCAGTT-3’ |
| Mouse IL-6 | 5’-ACCACTCCCAACAGACCTGTCT-3’ | 5’- CAGATTGTTTTCTGCAAGTGCAT-3’ |
| Mouse IL-10 | 5’-CAGAGAAGCATGGCCCAGAA-3’ | 5’-CACCTTGGTCTTGGAGCTTATTAAA-3’ |
| Mouse MCP-1 | 5’-CAGCAAGATGATCCCAATGAGTAG-3’ | 5’-TTTTTAATGTATGTCTGGACCCATTC-3’ |
